# Zinc-mediated carboxylations of allylic and propargylic halides in flow: synthesis of β-lactones *via* subsequent bromolactonization†

**DOI:** 10.1039/d2ra07715a

**Published:** 2023-01-24

**Authors:** Patrick J. Sutter, Guowei Kang, Sreekumar Vellalath, Daniel Romo

**Affiliations:** a Department of Chemistry and Biochemistry, Baylor University Waco 76798 Texas USA Daniel_Romo@baylor.edu; b Department of Chemistry, The Scripps Research Institute 10550 N. Torrey Pines Road La Jolla 92037 California USA

## Abstract

Zinc-mediated carboxylation of allylic halides under flow conditions delivered β,γ-unsaturated carboxylic acids and subsequent bromolactonization provides a streamlined process for the synthesis of γ-bromo-β-lactones. The described process further demonstrates the utility of organozinc reagents prepared by passage of allylic halides through a metallic zinc column integrated into a flow process. Use of a tube-in-tube reactor for efficient CO_2_ introduction led to improvements in conversion compared to a batch process and improved overall yields of β-lactones. The described flow process was also applied to propargylic bromides for the synthesis of allenic and propargylic acids.

## Abstract 2

Zinc-mediated carboxylation of allylic halides under flow conditions delivered β,γ-unsaturated carboxylic acids and subsequent bromolactonization provides a streamlined process for the synthesis of γ-bromo-β-lactones.

## Graphical abstract

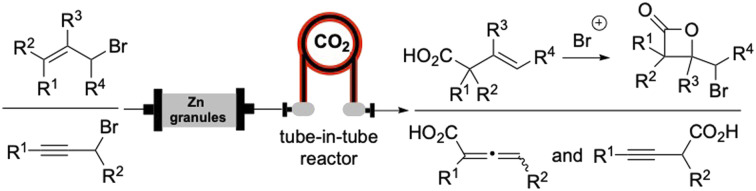

## Introduction

Organozinc reagents have been employed as mild carbon nucleophiles for the past 120 years.^1^ Compared to the analogous lithium or magnesium species, allylzinc reagents have greater stability, reduced basicity and excellent functional group tolerance, making them ideal alternatives when traditional metal-based carbon nucleophiles (*e.g.* Li(i), Mg(ii)) lead to undesired reactivity. The first reported conversion of an allylic halide to the corresponding allylzinc reagent was described by Gaudemar employing zinc dust.^2^ Several decades later, Knochel made seminal contributions to this area through use of LiCl-accelerated allylzinc formation which also reduces the propensity for dimerization through a Wurtz-type mechanism and improving solubility.^3,4^ Thus, LiCl has become a common additive in the generation and use of organozinc reagents for various reactions including 1,2-additions and metal-catalyzed cross-coupling reactions.^5^

**Scheme 1:**
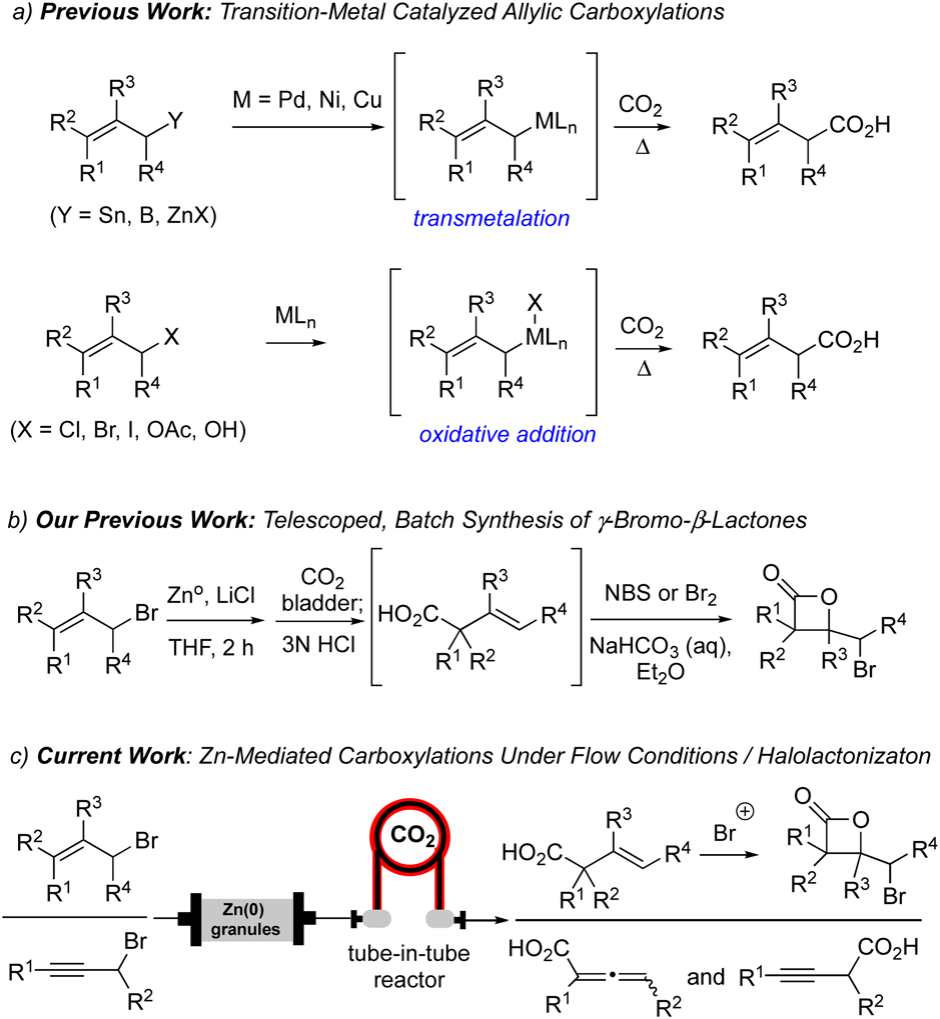
(a) Previous strategies for allylic carboxylation involving organometallic couplings *via* initial transmetalation or oxidative addition. (b) Our previous batch synthesis of γ-bromo-β-lactones through a telescoped process. (c) Current work employing generation of allyl and propargyl zinc reagents in flow and capture of CO_2_ through a tube-in-tube reactor.

Carbonyl compounds are common electrophiles employed with organozinc reagents, as described in seminal reports by Luche,^6^ Reformatsky,^7^ and Barbier.^1^ However, the use of CO_2_ has become increasingly explored as a C1 electrophile, particularly for allylic carboxylations.^8^ Previous methods for allylic carboxylation of aryl or alkyl zinc reagents employ Pd, Ni or Cu-based catalysts, high temperatures, and occasionally high-pressure conditions through transmetalation of allyl tin, boron, or zinc reagents (Scheme 1a).^9^ In addition, oxidative addition strategies of allylic halides, alcohols, and acetates for allylic carboxylation were described by Torii,^10^ Martin,^11^ Mita,^12^ and Nicholas.^13^ More recently, Mita reported a direct allylic C(sp^3^)–H carboxylation of allylarenes and 1,4-dienes, using a cobalt catalyst system with CO_2_ as a C1 building block, to generate terminal β,γ-unsaturated acids.^14^ Furthermore, the Ma group also demonstrated regioselective carboxylation of 2-alkynyl bromides with CO_2_ under batch conditions which was found to be dictated by the sterics of the allenyl *versus* propargylic zinc intermediates.^15^

Our group has a continued interest in developing concise methods for both the racemic and enantioselective synthesis of β-lactones.^16^ Recently, one strategy of keen interest to our group involves the net addition of CO_2_ to an alkene, likely one of the most direct routes to β-lactones. To date, we have developed two indirect ways to accomplish this challenging goal. The first route involves a Giese-type addition of CO_2_ radical anion to electron-poor alkenes followed by a halogenation-β-lactonization sequence.^17^ A second strategy, building on the work of Ma,^15^ made use of CO_2_ as a C1 electrophilic building block in the presence of allyl zinc reagents generated under mild batch conditions through a presumed electrophilic S_E_′ mechanism (Scheme 1b).^18^ This delivered β,γ-unsaturated acids in moderate yields which were then transformed to γ-bromo-β-lactones through subsequent γ-bromo-β-lactonizations. In the present study, we sought to improve the efficiency of this overall strategy to access β-lactones through use of a flow reactor using a Zn(0)-column organozinc generation and the use of a tube-in-tube reactor for increased CO_2_ introduction (Scheme 1c).

Some challenges for organozinc generation include the requirement of super-stoichiometric use of zinc dust, the generation of potential exotherms, and tedious filtration steps to collect the organometallic solution making this a labor-intensive process.^19^ Recent work by Alcazar described the on-demand and scalable production of organozinc reagents under a continuous flow protocol, utilizing a glass column filled with excess granular zinc metal integrated into a flow-reactor.^20^ This protocol allowed for reproducible concentrations of the organozinc reagent derived from ethyl bromoacetate which was used in subsequent Reformatsky reactions or Negishi couplings. Use of Zn(0) granules in a glass column removes the need for filtration of the generated organozinc reagent since unreacted Zn(0) remains in the column and can be used several times reducing waste. More precise temperature control of the glass column can be achieved using a heating jacket and a thermocouple in direct contact with the column. The polytetrafluoroethylene (PTFE) tubing also allows for efficient heat dispersion, an inherent advantage to flow reactors when performing reactions with potential for spontaneous exotherms.^21^

Building on these precedents, we envisioned a fully integrated flow system wherein allylic and propargylic halides could be transformed into the corresponding β,γ-unsaturated acids or allenic acids. Use of a Zn column in flow and a tube-in-tube reactor would enable efficient contact of gaseous CO_2_ with the reaction solution.^22^ Subsequent direct treatment with halogenating agents would enable a more streamlined strategy for the overall conversion of allylic and propargylic halides to γ-bromo-β-lactones.

## Results and discussion

### Flow module configuration

**Fig. 1:**
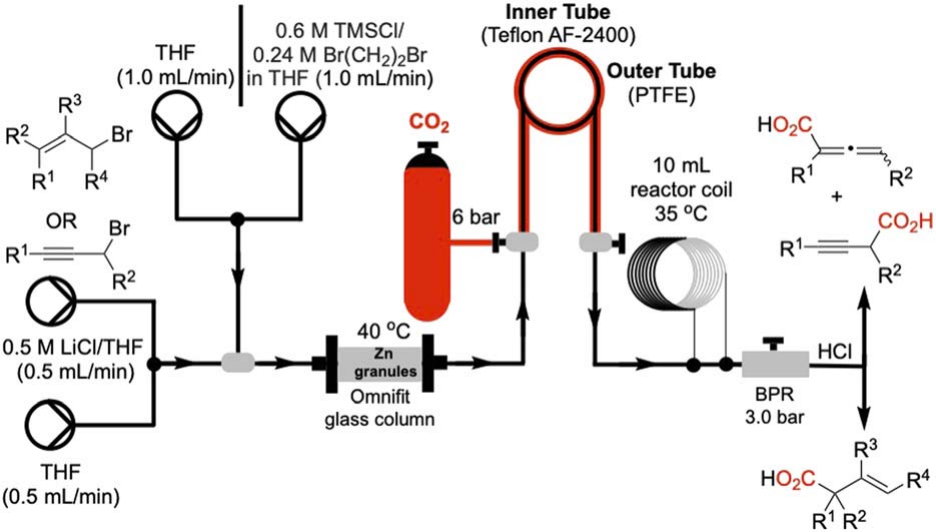
Schematic of the described Zn-mediated carboxylation reaction of allylic and propargylic halides. Flow system includes: (i) a glass column packed with granular zinc maintained at 40 °C (ii) CO_2_ (6 bar) introduction *via* a tube-in-tube reactor (inner tube: teflon AF-2400 permeable to CO_2_; outer tube: polytetrafluoroethylene (PTFE)) (iii) 10 mL coil reactor maintained at 35 °C and (iv) a back-pressure regulator (BPR) set to 3 bar. A final acidic quench (1 M HCl) delivers β,γ-unsaturated acids, allenic acids, and 3-alkynyl acids.

We utilized a flow system as shown schematically in Fig. 1. Granular zinc was dried by oven heating (125 °C, 2–3 h) and packed into an Omnifit glass column fitted with a heating jacket to maintain the temperature at 40 °C, and the column was connected in line *via* polytetrafluoroethylene (PTFE) tubing. Gaseous CO_2_ (6 bar) was directly connected to our tube-in-tube reactor,^22^ efficiently introducing gas into the liquid flow stream (substrate in THF, 0.66 M) leading to a homogeneous solution. A 10 mL heating-coil was maintained at 35 °C to facilitate the carboxylation. A back-pressure regulator maintained the internal pressure of the system at 3.0 bar. Prior to passing the solution of allyl or propargyl substrates through the Zn-column, the metal was activated by passing 5.0 mL of a THF solution of TMSCl (0.6 M) and 1,2-dibromoethane (0.24 M) at a flow rate of 1.0 mL min^−1^.^20^

### Optimization of flow protocol

We initiated our studies by first investigating various flow conditions for carboxylation of a solution of preformed allylzinc bromide prepared by our previously reported batch protocol.^18^ Using bromocyclohexene 1a as our test substrate, we examined different flow reaction variables including coil (reaction) temperature, flow rate, CO_2_ pressure, and back pressure regulation. After much experimentation, we determined that a temperature of 35 °C, a flow rate of 1.0 mL min^−1^, with 6.0 bar pressure of CO_2_ and 4.0 bar internal pressure gave a 50% yield of the desired acid 3a (see ESI for optimization studies†).

We next studied translation of this protocol to a complete flow process involving generation of the organozinc reagent employing the Zn(0)-column through adaptation of conditions reported by Alcazar.^20^ We subjected a solution of allylic bromide 1a (THF, 0.66 M) at a rate of 1.0 mL min^−1^ (*t*_R_ (residence time) = 3 min) through the Zn column and into the tube-in-tube reactor which gave the unsaturated acid 3a in 34% yield (Table 1, entry 1). We anticipated that increasing the residence time of the allylic bromide on the zinc column would increase conversion to the organozinc reagent 2a. This was confirmed by decreasing the flow rate to 0.5 mL min^−1^ (*t*_R_ = 6 min on Zn(0) column) resulting in a 53% yield of acid 3a (Table 1, entry 2). Reducing the concentration of the reaction to 0.33 M, by a factor of 2, was detrimental to the yield (33%, Table 1, entry 3). Further increases in residence time by decreasing flow to 0.25 mL min^−1^ (*t*_R_ = 12 min on Zn(0) column) did not lead to further improvements (Table 1, entry 4). Decreasing the internal pressure of CO_2_ from 4.0 to 3.0 bar while maintaining the external CO_2_ pressure at 6 bar, did not alter the yield significantly, (57%, Table 1, entry 5). Other solvents with greater CO_2_ solubility, namely CH_3_CN and DMF, led to very poor yield (<10%) likely due to lower conversion of the allylic bromide 1a to the organozinc reagent in these solvents (entries 6, 7). Building on Knochel's^4^ and Alcazar's studies,^4,20^ we prepared a solution of 2.3% LiCl in THF^23^ in efforts to accelerate organozinc formation, which led to a slight increase in yield to 61% (Table 1, entries 8). Ashfeld demonstrated improved conversion of alkyl and allylic halides to the corresponding organozinc reagents through use of catalytic amounts of TiCl_4_ for halide activation,^24^ however these conditions were incompatible in flow leading to clogging of the PTFE tubing perhaps due to the corrosive nature of HCl (entry 9). Finally, under optimized conditions, the flow process was scaled up to 20.0 mmol scale and purification was performed by acid–base extraction providing the highest yield of 71% (Table 1, entry 10).

**Table 1:** Optimization of continuous flow conditions^a^

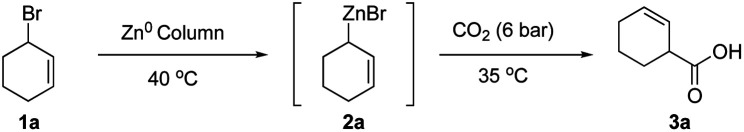					
Entry	Solvent	*t*_R_ (min)	Additives	Pressure (bar)	Yield^b^
1	THF	3	—	4.0	34
2	THF	6	—	4.0	53
3	THF^c^	6	—	4.0	33
4	THF	12	—	4.0	36
5	THF	6	—	3.0	57
6	MeCN	6	—	4.0	<10
7	DMF	6	—	4.0	<10
8	LiCl·THF	6	LiCl	3.0	61
9	LiCl·THF	6	TiCl_4_	3.0	<10
10	LiCl·THF	6	LiCl	3.0	71^d^

a See Fig. 1 for flow reaction setup. The Zn(0) column for allylzinc formation was maintained at 40 °C, the carboxylation following CO_2_ introduction in the tube-in-tube reactor is maintained at 35 °C in the reactor coil, and reactions were performed at 0.66 M allyl bromide concentration (flow rate: 0.25–1.0 mL min^−1^, *t*_R_ = 3–12 min on Zn(0) column as indicated).

b Yield refers to chromatographic purification (flash chromatography, SiO_2_).

c This reaction was performed at 0.33 M allylbromide concentration.

d The reaction was performed on 20.0 mmol scale and purification was performed through acid–base extraction (∼90–95% purity, ^1^H NMR).

### Allylic carboxylation scope

**Fig. 2:**
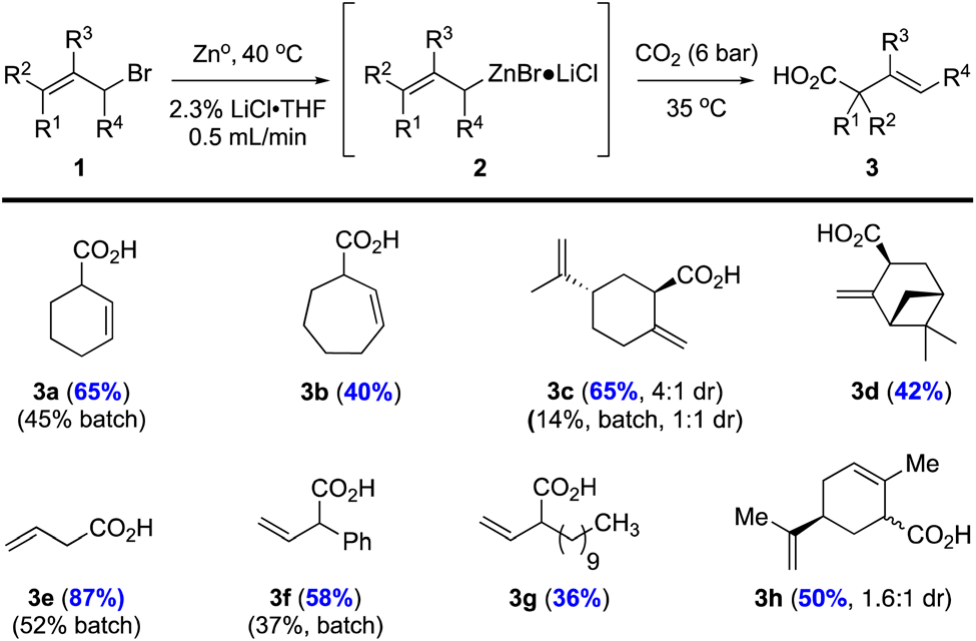
Carboxylation of various allylic bromides under flow conditions *versus* previously described batch conditions. ^a^Reactions were performed with allyl bromides 1 (1.0 equiv., 0.66 M) using the described, optimized flow conditions (see ESI for further details†) under 6 bar CO_2_ pressure (3 bar internal pressure). ^b^Yields for batch conditions *versus* flow methods (in blue) are provided. ^c^Yields refer to isolated products through acid–base extraction providing acids 3a–h in ∼90–95% purity (^1^H NMR).

With optimized conditions in hand, we conducted a limited substrate scope study including substrates that we previously utilized in a batch process^18^ for comparison (*i.e.* allylic bromides 1a, c, e, f) in addition to other allylic bromides not studied previously (*i.e.* allylic bromides 1b, d, g; Fig. 2). For all cases studied, our flow protocol gave higher isolated yields of acids 3a, c, e, and f with increases of ∼20–50% with the most dramatic improvement being the perillyl alcohol-derived carboxylic acid 3c (65% yield, 4 : 1 dr *vs.* 14%, 1 : 1 dr in batch). The increase in diastereoselectivity could be attributed to more efficient heat dispersion under flow conditions. All acids were isolated through acid–base extraction leading to products that were 90–95% pure as judged by ^1^H NMR. The extended aliphatic acid 3g was isolated in modest yield (36%), likely due to its inherent lipophilic nature and reduced solubility in THF. Cycloheptene acid 3b was isolated in 40% yield, while the myrtenol derivative 3d was isolated as a single diastereomer (>19 : 1 dr) in 42% yield. The carvone derived allylic bromide 1h (6 : 1 dr) delivered acid 3h in 50% yield, as a 1.6 : 1 inseparable mixture of diastereomers. The observed stereochemical erosion can be attributed to a non-stereospecific organozinc formation process or a Schlenk equilibrium as noted previously by Knochel.^25^

### Bromo-β-lactonization

**Fig. 3:**
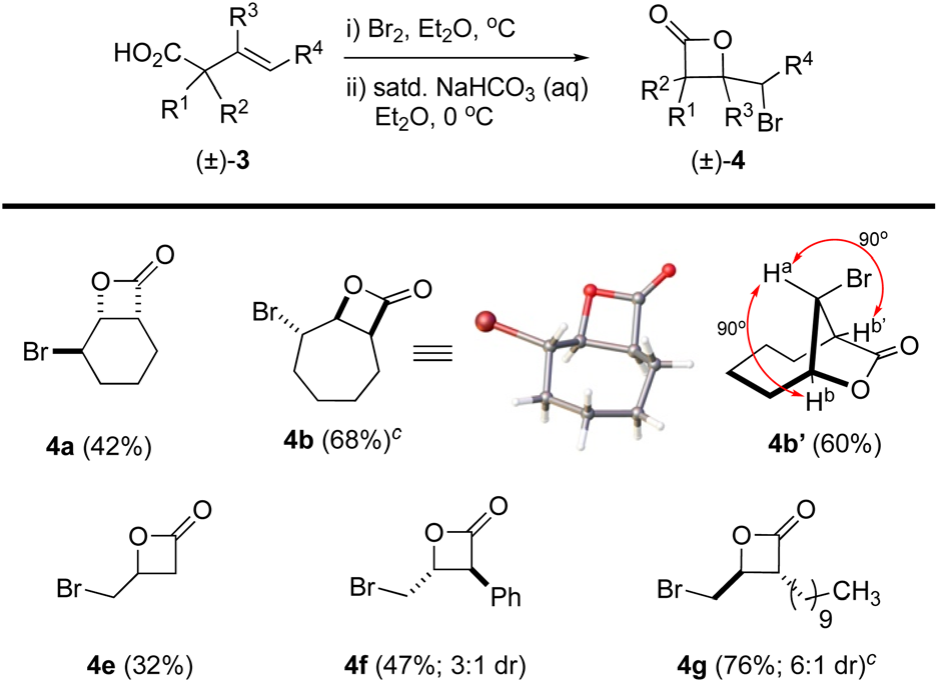
Synthesis of γ-bromo-β-lactones 4*via* bromo-lactonization of carboxylic acids 3. ^a^Reactions were performed with isolated acids 3, Br_2_ (1.5 equiv.), saturated NaHCO_3_ (aq) in Et_2_O or CH_2_Cl_2_, at 0 °C. ^b^Yields refer to purified (flash chromatography, SiO_2_) β-lactones 4a–g as a mixture (as indicated) or as single diastereomers (>19 : 1, 600 MHz ^1^H NMR). ^c^Reaction was performed using NBS (1.5 equiv.) in CH_2_Cl_2_ at 0 °C. (Inset: X-ray structure of β-lactone 4b).

The β,γ-unsaturated acids 3, obtained through acid–base extraction, were next directly subjected to bromolactonization conditions using a biphasic reaction mixture of either Br_2_ in Et_2_O or NBS in CH_2_Cl_2_ with saturated, aqueous NaHCO_3_ to deliver γ-bromo-β-lactones 4 (Fig. 3).^18^ This avoided purification of the polar carboxylic acids 3 by silica gel chromatography which led to loss of material. Bicyclic β-lactones 4a and 4b were isolated in 42% and 68% yield, respectively, from acids 3a and 3b. β-Lactone 4b was found to be crystalline, allowing for X-ray analysis (see SI). However, slow conformational interconversion of β-lactone 4b led to very broad peaks in the ^1^H NMR. For complete structural conformation of β-lactone 4b, we performed variable temperature (VT) ^1^H-NMR studies and optimal conformational exchange was observed at 70 °C (see ESI for further details†). Interestingly, when acid 3b was treated with Br_2_ in Et_2_O/aq. NaHCO_3_, the expected bicyclic β-lactone 4b was not isolated, but instead gave the [4.2.1] bridged bicyclic lactone 4b′ (∼60%). The lack of *J*_a,b/b′_ coupling was highly indicative of this bridged compound based on models which showed an ∼90° dihedral angle. The perillyl acid derivative 3c underwent non-regioselective bromination when treated with Br_2_, yielding a complex mixture of products. However, it can be accessed using NBS and catalytic amounts of (DHQD)_2_PHAL as we previously described.^26^ Lastly, the myrtenol acid derivative 3d led to a complex mixture of products presumably due to the inherent ring strain upon formation of the tricyclic product. The linear aliphatic acids 3e, 3f, and 3g provided the corresponding β-lactones 4e (volatile), 4f, and 4g in 32, 47 (3 : 1 dr), and 76% (6 : 1 dr) yields, respectively.

### Carboxylation of propargyl-halides in flow

**Fig. 4:**
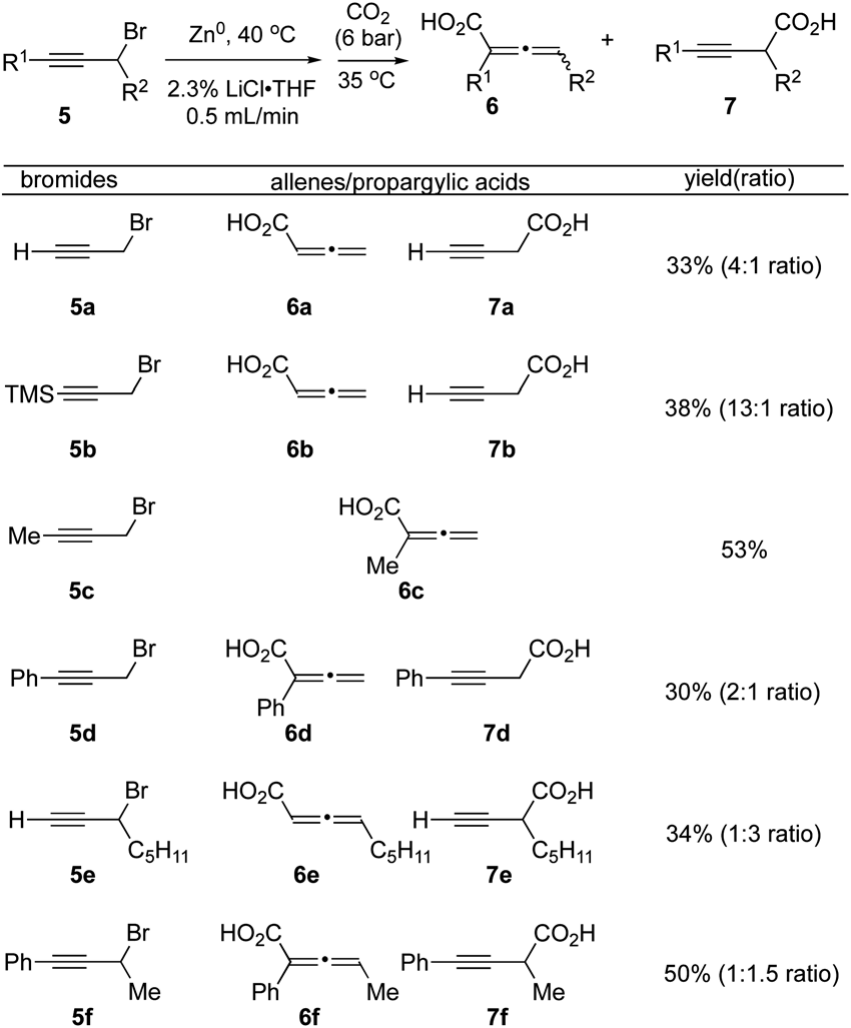
Synthesis of allenes 6 and propargylic acids 7 under flow conditions. ^a^Reactions were performed with propargyl bromides 5 (1.0 equiv., 0.66 M) using optimized flow conditions. ^b^Yields refer to isolated (acid–base extraction) allenic/propargylic acids.

We next applied our optimized flow protocol to the carboxylation of a collection of primary and secondary propargylic bromides building on previous work by Ma.^15^ High regioselectivity was dependent on the substitution patterns of propargyl bromide substrates, along with use of DME as a chelating solvent (Fig. 4). We opted to continue utilizing THF for its ease of handling and drying. Propargyl bromide 5a underwent carboxylation to deliver a mixture of the allenic acid 6a and propargyl acid 7a in 33% yield (4 : 1 inseparable mixture). The silyl protected alkyne 5b tolerated the flow conditions, however the silyl group was cleaved during acidic workup, to provide allenic and propargylic acids 6b and 7b with increased selectivity for the allene acid 6b (13 : 1). The methyl alkyne 5c delivered only the allene acid 6c in 53% yield. Phenyl alkyne 5d produced both allene and propargylic acids 6d and 7d in a 2 : 1 ratio (30% yield). The secondary propargylic bromide 5e gave a 1 : 3 ratio of allene and propargylic acids 6e and 7e (34%). Finally, the phenyl alkyne, secondary bromide 5f produced a near equimolar mixture of allene 6f and propargylic acid 7f (1 : 1.5, 50% yield).

### Bromo-γ-lactonization of an alkynyl acid

**Scheme 2:**
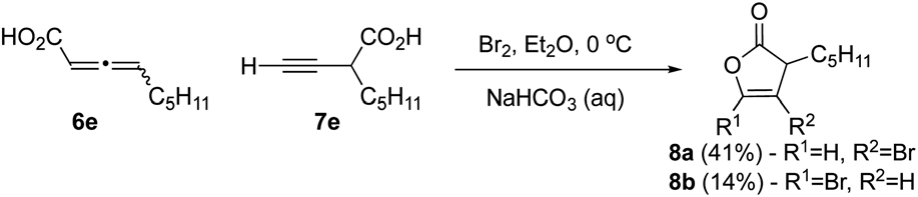
Bromolactonization of alkynyl substrates.

We next studied bromination of the propargylic acid 7e and allenic acid 6e (3 : 1 ratio), to determine the regioselectivity of bromolactonization. Similar transformations using iodine were previously reported by Larock,^27^ with an array of substituted propargyl acids and thus our expectation was that only the propargylic acid would react. When carboxylic acids 6e and 7e were subjected to bromolactonization conditions (Scheme 2), this led to the exclusive generation of furanones 8a and 8b*via* the favorable 5-*endo*-dig cyclization in accord with results by Larock in 55% combined yield. No evidence for β-lactone formation was observed except by crude IR rather only the butenolides 8a,b could be isolated.

## Conclusions

In summary, we developed a flow process for conversion of allylic bromides into their corresponding β,γ-unsaturated acids building on foundational work by Alcazar.^20^ Further, we developed a streamlined, telescoped approach for converting allylic bromides into γ-bromo-β-lactones through a subsequent bromolactonization that reduces reaction time and amount of Zn(0) used. The process involves enhanced allyl zinc bromide formation in flow, efficient delivery of CO_2_ through use of a tube-in-tube reactor, and subsequent rapid bromolactonization. The reaction was performed up to 20.0 mmol scale, however the described method utilizes commercially available materials and can theoretically be scaled up to greater than 100 mmol scale limited only by the size and volume of the Zn(0) column used. We also demonstrated that the optimized carboxylation process in flow applied to propargylic bromides allows access to allenic and propargylic acids. While these are often produced as mixtures, this provides a rapid synthesis of allenes which could presumably be isolated following subsequent reactions (*e.g.* bis-alkylation of the propargylic acids with mono-alkylation of the allenic acid). Subsequent bromolactonization of one of these mixtures 6e/7e under basic conditions delivered the expected 5-*endo*-dig cyclization leading to regioisomeric bromobutenolides. While the described methods generally provide moderate to good yields, the described flow procedure enables a scalable process for the production of β-lactones from simple, commercially available allylic halides and also mixtures of allenic acids and homopropargylic acids, which could likely be separated after further functionalization, from readily available propargylic bromides.

## Author contributions

PS performed optimization studies and substrate scope studies; GK and SV performed preliminary optimization studies and offered advice throughout the project. All authors were involved in writing the manuscript and have given approval for the final version.

## Conflicts of interest

There are no conflicts to declare.

## Supplementary Material

RA-013-D2RA07715A-s001

RA-013-D2RA07715A-s002
