# Supplementary material for: Zinc-mediated carboxylations of allylic and propargylic halides in flow: synthesis of β-lactones via subsequent bromolactonization
Source: RSC Adv. 2023 Jan 24;13(6):3468–73. doi: 10.1039/d2ra07715a (PMC9871729; doi:10.1039/d2ra07715a)
Supplement: RA-013-D2RA07715A-s001 [file RA-013-D2RA07715A-s001.pdf]

Electronic Supplementary Material (ESI) for RSC Advances.
This journal is © The Royal Society of Chemistry 2023

Zinc-Mediated Carboxylations of Allylic and Propargylic Halides in Flow: Synthesis of β-Lactones via Subsequent Bromolactonization

Patrick Sutter, Guowei Kang, Sreekumar Vellalath, and Daniel Romo*

Department of Chemistry and Biochemistry, Baylor University, One Bear Place #97348, Waco, TX 76798, United States

Table of Contents

General Information	2
Optimization Studies	3
Experimentals	5
^{1}H and ^{13}C NMR Spectra	24
X-Ray Crystallographic Data	34

General Information

All non-aqueous reactions were performed under an inert atmosphere of nitrogen or argon in oven-dried glassware. All reaction solvents used were dried by passing through activated molecular sieves or alumina (solvent purification system). Unless otherwise indicated, all reagents were used as received from commercially available sources. Deuterated solvents were purchased from either Aldrich or Cambridge Isotopes and used as received. ^{1}H NMR spectra were measured at 600 MHz or 500 MHz and referenced relative to residual chloroform (7.26 ppm) and reported in parts per million. Coupling constants (J) were reported in Hertz (Hz), with multiplicity reported following usual convention: s, singlet; d, doublet; t, triplet; q, quartet; dd, doublet of doublets; ddd, doublet of doublet of doublets; td, triplet of doublets; qd, quartet of doublets; dt, doublet of triplets; dq, doublet of quartets; m, multiplet; br, broad signal; app, apparent. ^{13}C NMR spectra were measured at 150 MHz and 126 MHz and referenced relative to residual chloroform (77.16 ppm) and was reported in parts per million (ppm). Flash column chromatography was performed with 60Å Silica Gel (230-400 mesh) as stationary phase using a gradient solvent system or on an automated flash chromatography system using SiO_2 cartridges (50 μm particle size) unless noted otherwise. High resolution mass spectra (ESI) were obtained with a Thermo Orbitrap Discovery system. Thin Layer Chromatography (TLC) was performed using glass-backed silica gel F254 (Silicycle, 250 μm thickness). Visualization of developed plates was performed by fluorescence quenching, potassium permanganate staining, or Iodine staining. Fourier Transform Infrared (FTIR) spectra were recorded as thin films on NaCl plates. The RS-200 flow system, Omnifit Glass Column and gastropod (tube-in-tube reactor) used in this methodology were all purchased from Vapourtec. A back-pressure regulator (Zaiput BPR-10) was utilized with the Vapourtec system. Allylic bromides, unless otherwise stated, were purchased from Sigma-Aldrich, Acros, and Oakwood. Bromine was purchased from Acros. Granular Zinc (20-30 mesh) was purchased from Sigma-Aldrich and CO_2 (99.99%) was purchased from Praxair/Linde.

Abbreviation List

THF – tetrahydrofuran	TMSCl – trimethylsilyl chloride
PTFE - polytetrafluoroethylene	TLC – thin layer chromatography
NBS – *N*-bromosuccinimide	UV – ultraviolet lamp
EtOAc – ethyl acetate	H-NMR – proton nuclear magnetic resonance
Et_2O – diethyl ether	BPR – back pressure regulator

Optimization Studies

Batch Preparation of AllylZinc Halide Solution and Carboxylations in Flow (Table S1)

Table S1. Optimization of carboxylations (in flow) of preformed allylzinc halide solution

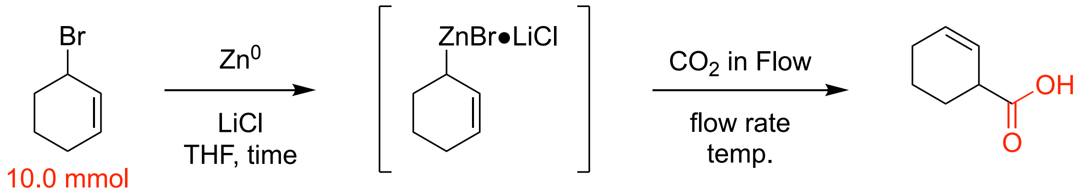

entry	Zn drying method	time	coil temp.	flow rate	yield (%)
1	heat gun 15 min	2 h	35	0.5 mL/min	35
2	none	2 h	"	"	28
3	oven 16 h	3 h	"	"	45
4	oven 3 h	4 h	"	"	44
5	"	2.5 h	55	"	45
6	"	3 h	75	"	40
7	"	"	25	"	37
8	"	"	35	1.0 mL/min	50
9	"	"	"	2.0 mL/min	35
10	"	"	45	"	38

Note: After the organozinc formation was complete (2-4 hours), the THF solution was filtered away from the excess zinc metal using a 0.45 μm PTFE syringe filter (VWR catalog 28145-497) and then transferred to a 25 mL pear-shaped flask.

Organozinc Formation and Carboxylations in Flow (Table S2)

Table S2. Additional optimization experiments for the streamlined flow procedure

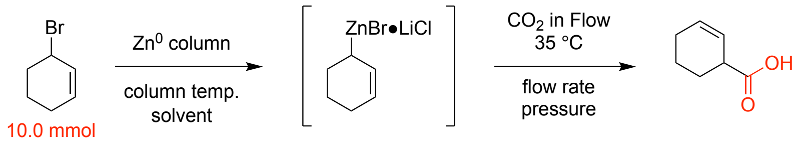

entry	solvent	conc. [M]	Zn drying method	column temp.	flow rate	additives	pressure	yield (%)
1	THF	0.66	none	40 °C	0.5 mL/min	-	3 bar	40
2	THF	0.66	none	40 °C	0.25 mL/min	-	4 bar	26
3	THF	0.33	none	40 °C	1.0 mL/min	-	4 bar	34
4	THF	0.66	oven 3 h	40 °C	0.5 mL/min	-	3 bar	53
5	THF	0.33	oven 3 h	40 °C	0.5 mL/min	-	4 bar	33
6	THF	0.66	oven 3 h	40 °C	0.25 mL/min	-	4 bar	36
7	MeCN	0.66	oven 3 h	40 °C	0.5 mL/min	-	4 bar	<10
8	DMF	0.66	oven 3 h	40 °C	0.5 mL/min	-	4 bar	trace
9	THF	0.66	oven 3 h	40 °C	0.5 mL/min	-	3.5 bar	34
10	2.3% LiCl•THF	0.66	oven 3 h	40 °C	0.5 mL/min	LiCl sol.	3 bar	58
11	THF	0.66	oven 3 h	40 °C	0.5 mL/min	Zn/LiCl column	3 bar	52
12	2.3% LiCl•THF	0.66	oven 3 h	60 °C	0.5 mL/min	LiCl sol.	3 bar	18
13	2.3% LiCl•THF	0.66	oven 3 h	50 °C	0.5 mL/min	LiCl sol.	3 bar	26
14	2.3% LiCl•THF	0.66	oven 3 h	30 °C	0.5 mL/min	LiCl sol.	3 bar	44
15	2.3% LiCl•THF	0.66	oven 3 h	20 °C	0.5 mL/min	LiCl sol.	3 bar	61
16	2.3% LiCl•THF	0.66	oven 3 h	40 °C	0.5 mL/min	Ti (20 mol %)	3 bar	8
17	2.3% LiCl•THF	0.66	oven 3 h	40 °C	0.5 mL/min	LiCl sol.	3 bar	57
18*	2.3% LiCl•THF	0.66	oven 3 h	40 °C	0.5 mL/min	LiCl sol.	3 bar	71*

Note: *20.0 mmol scale; yield refers to product isolated by an acid-base extraction procedure to avoid loss of polar carboxylic acid on silica gel.

Experimental Procedures

Titration of Allylzinc Bromide Solution (Table S3)

Note: Titration studies were performed to determine how many times a packed zinc-column could be used. Titrations were carried out following a known procedure described by Alcazar using iodine.[1] A solution of the simple allylzinc bromide was generated and collected in an oven-dried flask under argon following the protocol outlined by Alcazar.[1]

Table S3. Additional optimization experiments for the streamlined flow process for allylzinc halide formation with a Zn(0) column

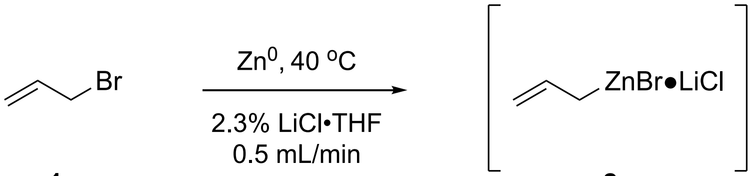

1e 2e

$$\text{Concentration [M] of allylzinc bromide} = \frac{\text{Iodine (mg)}}{(\text{MW Iodine})(\text{vol RZnBr})}$$

Entry	W of Iodine (mg)	Vol RZnBr (mL)	Concentration [M]	% Conversion
1	12.3	1.25	0.03877	43%
2	14.3	1.26	0.04472	48%
3	14.5	1.47	0.03886	41%
4	14.6	1.05	0.05478	58%
5	10.4	7.95	0.005154	6%

Zinc column preparation: 9.9-10.1 g of zinc metal was dried in an oven for 1 h (125 °C) and loaded into an Omnifit glass column (3.42 mm x 90.0 mm). The column is activated by flowing through a 0.6 M TMSCl/0.24 M $C_2H_4Br_2$ solution in THF (5 mL), and 5.0 mmol of allyl bromide in 2.3% LiCl•THF (0.66 M) was pumped through the activated column. The collected allylzinc bromide solution was titrated against I_2 to determine its concentration [M] and % conversion. From the titration study above, we recommend replacing the zinc metal in the glass column after every 3 to 4 experiments to avoid significant decreases in yields.

Preparation of Starting Material Bromides

Note: Unless listed below, all starting material bromides were purchased from commercial vendors.

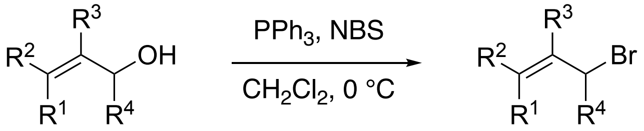

Standard Appel Procedure: To an oven dried 100mL round-bottomed flask equipped with a stir bar was added 1.57 g (6.00 mmol) of triphenylphosphine and 25 mL of dichloromethane. The solution was then cooled to 0 ℃ before the addition of dry, recrystallized *N*-bromosuccinimide (NBS) (1.07 g, 6.00 mmol), after which the colorless solution turned maroon in color. The mixture was stirred for 30 min and 5.00 mmols of an allylic or propargylic alcohol was then added to the maroon solution and monitored by TLC (10% EtOAc:hexanes, UV or $KMnO_4$). Once judged complete by TLC, the reaction solution was diluted with a 10% Et_2O:hexanes solution to precipitate phosphine oxide. The reaction solution was filtered through a short, fat pad of silica gel, washing with additional 10% Et_2O:hexanes. The filtrate was then concentrated under reduced pressure. TLC and H-NMR was used to determine if additional purification was needed. If so, flash chromatography (SiO_2, 10%EtOAc:hexanes eluent) delivered the desired allylic or propargylic bromide as a colorless to off-yellow oil.

3-bromocyclohept-1-ene (1b): Prepared following the standard Appel conditions described above using cyclohept-2-en-1-ol (1.35 mL, 12.0 mmol), triphenylphosphine (3.46 g, 13.2 mmol), and NBS (2.35 g, 13.2 mmol), delivering the allylic bromide as a colorless oil (1.07 g, 6.12 mmol, 51% yield). Spectral data matched that previously reported.[2]

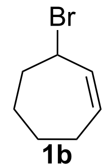

1b

(*S*)-1-(bromomethyl)-4-(prop-1-en-2-yl)cyclohex-1-ene (1c): Prepared following the standard Appel conditions described above using (*S*)-perillyl alcohol (1.90 mL, 12.0 mmol), triphenylphosphine (3.46 g, 13.2 mmol), and NBS (2.35 g, 13.2 mmol), delivering the allylic bromide as a colorless oil (1.39 g, 6.48 mmol, 54% yield). Spectral data matched that previously reported.[3]

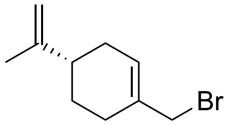

1c

(1*R*,5*S*)-2-(bromomethyl)-6,6-dimethylbicyclo[3.1.1]hept-2-ene (1d): Prepared

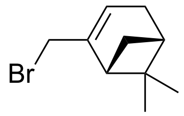

1d

following the standard Appel conditions described above using (–)-myrtenol (1.85 mL, 12.0 mmol), triphenylphosphine (3.46 g, 13.2 mmol), and NBS (2.35 g, 13.2 mmol), delivering the allylic bromide as a colorless oil (2.40 g, 11.2 mmol, 93% yield). Spectral data matched that previously reported.[4]

(*E*)-1-bromotridec-2-ene (1g): Prepared following the standard Appel conditions

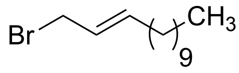

1g

described above using (*E*)-tridec-2-en-1-ol (2.60 mL, 12.0 mmol), triphenylphosphine (3.46 g, 13.2 mmol), and NBS (2.35 g, 13.2 mmol), delivering the allylic bromide as a colorless oil (2.76 g, 10.6 mmol, 88% yield). Spectral data matched that previously reported.[5]

(3-bromoprop-1-yn-1-yl)benzene (5d): Prepared following the standard Appel

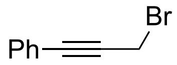

5d

conditions described above using 3-phenylprop-2-yn-1-ol (950 μL, 7.62 mmol), triphenylphosphine (2.40 g, 9.15 mmol), and NBS (1.63 g, 9.15 mmol), delivering the propargylic bromide as a colorless oil (946 mg, 4.85 mmol, 64% yield). Spectral data matched that previously reported.[6]

3-bromooct-1-yne (5e): Prepared following the standard Appel conditions described

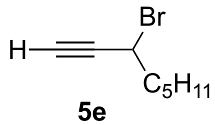

5e

above using oct-1-yn-3-ol (1.45 mL, 10.0 mmol), triphenylphosphine (3.15 g, 12.0 mmol), and NBS (2.14 g, 12.0 mmol), delivering the propargylic bromide as a colorless oil (1.33 g, 7.02 mmol, 70% yield). Spectral data matched that previously reported.[7]

(3-bromobut-1-yn-1-yl)benzene (5f): Prepared following the standard Appel conditions

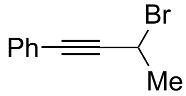

5f

described above using 4-phenylbut-3-yn-2-ol (2.75 mL, 20.0 mmol), triphenylphosphine (6.82 g, 26.0 mmol), and CBr_4 (7.96 g, 24.0 mmol), delivering the product as an orange oil (2.50 g, 12.0 mmol, 60% yield). Spectral data matched that previously reported.[8]

Synthesis of carvone bromide 1h

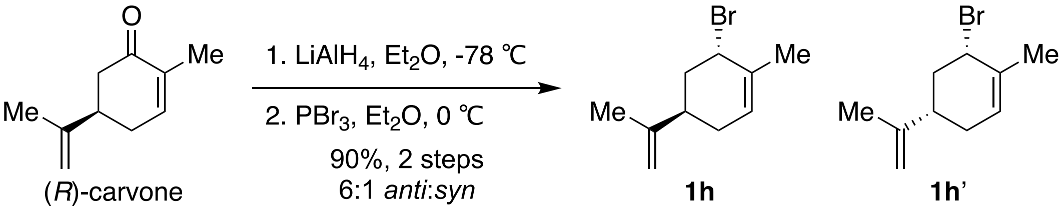

6-bromo-1-methyl-4-(prop-1-en-2-yl)cyclohex-1-ene (1h): To an oven-dried, 500 mL round-bottomed flask, a stir bar and solid $LiAlH_4$ (~1.5 g; 3 reagent grade 95% pellets, each weighing ~0.5 g; 39.5 mmol) was added before being fitted with a septum and purged with inert gas (argon or nitrogen). After 5-10 min of purging, the solid was dissolved in Et_2O (90 mL) and stirred for 30 mins until a uniform, grey suspension was observed. The reagent mixture was cooled to -78 °C before the slow, dropwise addition of (*R*)-carvone (6.20 mL, 39.5 mmol). The reaction mixture stirred for 30 min at -78 °C, after which the excess $LiAlH_4$ was carefully quenched by slow addition of a 10% H_2SO_4 solution (30 mL) added dropwise at -78 °C (flask was opened to air with nitrogen flowing through to avoid pressure build up and allow the release of H_2). After complete addition of the H_2SO_4 solution and no further gas evolution, the reaction mixture was gradually warmed to 0 °C, and treated with a saturated solution of Rochelle's salt (potassium sodium tartrate, 30 mL). The biphasic mixture was vigorously stirred for 2 h until the layers were no longer cloudy and the mixture was transferred to a 500 mL separatory funnel where the two layers were separated. The aqueous layer was extracted using Et_2O (3 x 50 mL), and the combined organic layers were washed with brine (1 x 50 mL), dried over Na_2SO_4, filtered, and concentrated under reduced pressure to deliver ((–)-*cis*) carveol as an off yellow oil (99%, >19:1 dr) which was used without further purification. Spectral data matched that previously reported.[20]

To an oven-dried, 250 mL round-bottomed flask equipped with a stir bar was added ((–)-*cis*) carveol (2.4 mL, 15.0 mmol) before being fitted with a septum and purged with inert gas (argon or nitrogen). After 5-10 mins of purging, the starting material was dissolved in Et_2O (60 mL) and cooled to 0 °C, before being treated with PBr_3 (1.4 mL, 15.0 mmol) (*Note: standard Appel conditions led to complex mixtures of products, leading to the eventual use of PBr_3). The reaction was monitored by TLC (10% EtOAc:hexanes, $KMnO_4$) stirring for 1 h before being quenched with a saturated solution of $NaHCO_3$ (30 mL) added

dropwise at 0 °C (flask was opened to air with nitrogen flowing through to avoid pressure build up and allow the release of gas). The biphasic solution was then transferred to a 250 mL separatory funnel and the two layers were separated. The organic layer was further washed with $NaHCO_3$ (1 x 30 mL), brine (1 x 30 mL), dried over $MgSO_4$, filtered, and concentrated under reduced pressure to deliver an off-yellow oil. The bromide **1h** was found to be unstable on various forms of chromatographic stationary phases (treated/untreated silica, basic/neutral alumina, and fluorosil) and thus was used directly in the next step without any further purification (2.91 g, 13.5 mmol, 90% yield, 6:1 dr). Compound **1h** was previously reported without spectral data[21] (crude NMR data included below). However, the authors determined the *anti*-bromide isomer to be the major diastereomer following subsequent S_n2 vs S_n2' transformations which were found to competitive mechanistic pathways (as shown at right).

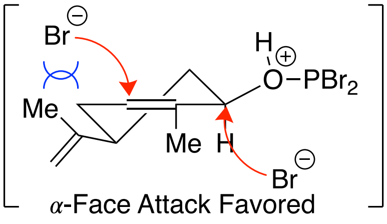

TLC (10% EtOAc:hexanes, $KMnO_4$), R_f = 0.82

^{1}H NMR (600 MHz, $CDCl_3$, major diastereomer) δ 5.63 (dd, *J* = 5.5, 1.4 Hz, 1H), 4.80 – 4.75 (m, 1H), 4.76 – 4.71 (m, 2H), 2.72 – 2.63 (m, 1H), 2.32 – 2.30 (m, 1H), 2.29 – 2.28 (m, 1H), 2.08 – 2.01 (m, 1H), 1.94 (ddd, *J* = 14.5, 12.5, 4.0 Hz, 1H), 1.82 (dt, *J* = 2.8, 1.5 Hz, 3H), 1.76 (s, 3H).

^{13}C NMR (151 MHz, $CDCl_3$, major diastereomer) δ 148.5, 134.2, 127.0, 109.5, 55.6, 37.8, 35.7, 31.0, 21.7, 21.1.

Zinc-Mediated Carboxylation Flow Reaction Setup: Schematic, Picture, and Description

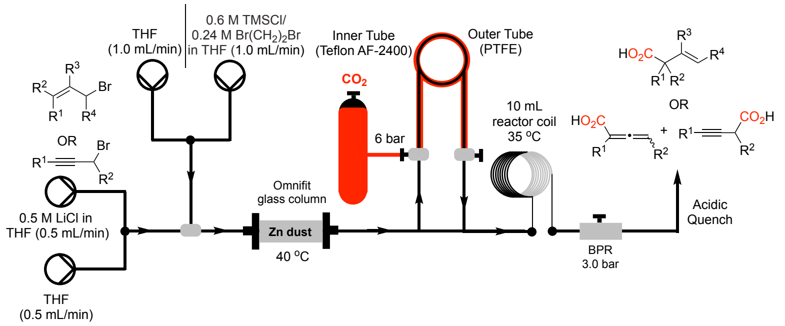

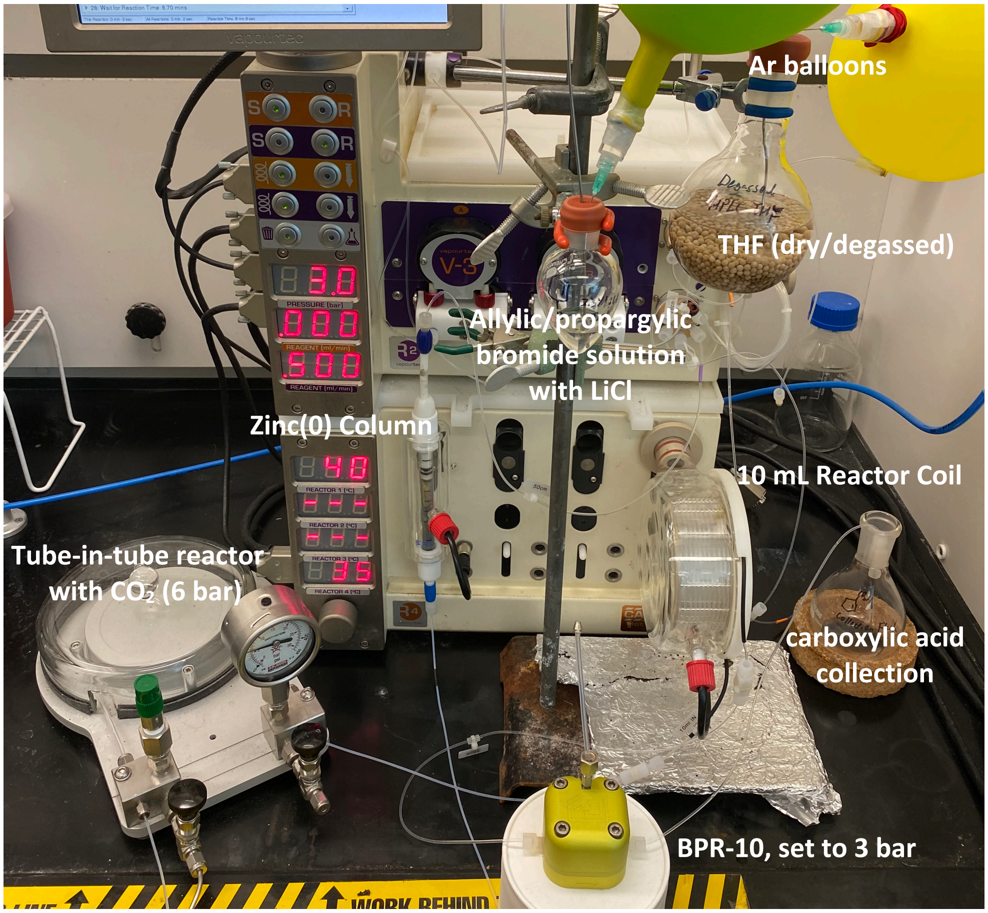

The Vapourtec flow system was equipped with an Ominfit glass column, a tube-in-tube (Gastropod) reactor, a CO_2 inlet (6 bar), a 10 mL heated reactor coil (35 °C), and a back-pressure regulator (Zaiput BPR-10, 3 bar). The glass column of metallic zinc was prepared and activated as described above using a solution of TMSCl and 1,2-dibromoethane. The reaction mixture containing the allylic/propargylic bromide substrate was maintained under an argon atmosphere and dissolved in a 2.3% LiCl•THF solution (prepared following a known procedure[9]). The allyl/propargyl bromide solution is drawn into the flow system under argon using perfluoroethylene tubing fitted with a needle through the zinc column and the tube-in-tube (CO_2) reactor leading to the desired carboxylic acids.

Representative Procedure for Zinc-Mediated Allylic Carboxylation in Flow as described for β,γ-unsaturated acid 3a

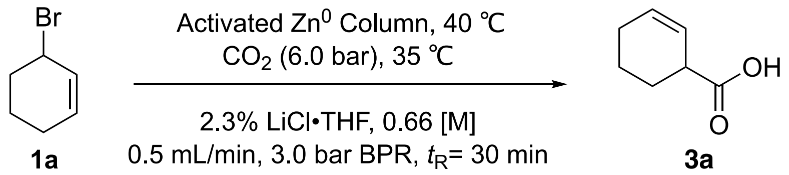

Cyclohex-2-ene-1-carboxylic acid (3a): An oven-dried 50 mL pear shaped flask was charged with bromide **1a** (2.30 mL, 20.0 mmol; passed through plug of alumina until colorless), then dissolved in 30 mL of a 2.3% LiCl•THF solution. The reaction flask was purged with a balloon filled with argon for 15 min. The solution was then drawn into the Vapourtec R2 through a fluoroethylene polymer (FEP) tubing with an attached needle. The reaction run is then initiated with a pre-set flow rate of 0.5 mL/min. The reaction mixture is pumped into a 5 mL glass column packed with granular zinc metal (maintained at 40 °C) to form the reactive organozinc species. The reaction mixture is then pumped into a 1.2 mL tube-in-tube (under 6 bar CO_2 pressure) reactor (Gastropod®) to form a homogeneous solution with dissolved CO_2. The solution then enters a 10 mL heated reactor coil (maintained at 35 °C) to facilitate the carboxylation. Once 30.0 mL (volume set in the instrument) of the reaction mixture is pumped through, the input was switched to a reservoir of THF and 32.7 mL (calculated automatically by the flow instrument based on tubing length employed) is used to push the reaction mixture through the flow reactor. A backpressure regulator (set to 3 bar) after the 10 mL reactor coil prevents out-gassing of dissolved CO_2 from THF in the reactor. The reaction mixture was collected into a 250 mL round-bottomed flask containing 20 mL of 1 M HCl. After all effluent was collected, the flask was briefly swirled, and the reaction solution was concentrated under reduced pressure. The flask's contents were transferred to a 125 mL separatory funnel and extracted with diethyl ether (3 x 20 mL). The combined organics were then washed with 1 M NaOH (3 x 10 mL) and the layers were separated. The combined basic aqueous washes were acidified to pH ≤ 3 using 1 M HCl and then extracted with diethyl ether (3 x 30 mL) to isolate the carboxylic acid **3a**. The combined organics were washed with brine (1 x 30 mL), dried over Na_2SO_4, filtered, and concentrated under reduced pressure to

deliver carboxylic acid **3a** as a colorless oil (1.62 g, 12.9 mmol, 71% yield). Spectral data matched that previously reported.[10]

Note: The yield for all reactions was calculated based on the amount of substrate actually used in the reaction, which is determined by taking into account the 0.65 mL of solution that remains in the tubing between the flask and the joint connector, and taking into account the volume of unused bromide remaining in the reaction flask. The total amount (in mmol) of unused bromide is then accounted for when calculating the yield for each reaction based on the equation below.

$$\text{mmol used in experiment} = \frac{\text{input vol Br sol} - \text{inlet tubing vol}}{\text{total vol Br sol}}\ x\ calculated\ mmol$$

Cyclohept-2-ene-1-carboxylic acid (3b): Prepared according to the representative procedure using 3-bromocyclohept-1-ene **1b** (630 μL, 4.39 mmol) to deliver carboxylic acid **3b** as a colorless oil (196 mg, 1.40 mmol, 43% yield). Spectral data matched that previously reported.[11]

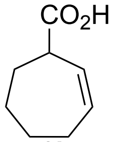

3b

(1*R*,5*S*)-2-Methylene-5-(prop-1-en-2-yl)cyclohexane-1-carboxylic acid (3c): Prepared according to the representative procedure using (*S*)-1-(bromomethyl)-4-(prop-1-en-2-yl)cyclohex-1-ene **1c** (1.50 mL, 8.44 mmol) to deliver carboxylic acid **3c** as a colorless oil (785 mg, 4.36 mmol, 65% yield, 4:1 dr). Spectral data matched that previously reported.[3]

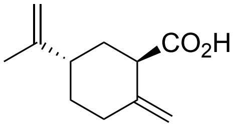

3c

(1R,3S,5R)-6,6-Dimethyl-2-methylenebicyclo[3.1.1]heptane-3-carboxylic acid (3d): Prepared according to the representative procedure using (1*R*,5*S*)-2-(bromomethyl)-6,6-dimethylbicyclo[3.1.1]hept-2-ene **1d** (1.70 mL, 10.0 mmol) to deliver the carboxylic acid **3d** as a colorless oil (607 mg, 3.37 mmol, 42% yield). Spectral data matched that previously reported.[11]

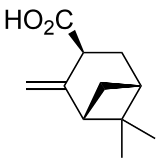

3d

But-3-enoic acid (3e): Prepared according to the representative procedure using 3-bromoprop-1-ene **1e** (850 μL, 10.0 mmol) to deliver carboxylic acid **3e** as a colorless oil (607 mg, 7.03 mmol, 87% yield). Spectral data matched that previously reported[10].

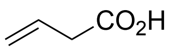

3e

2-Phenylbut-3-enoic acid (3f): Prepared according to the representative procedure using (*E*)-(3-bromoprop-1-en-1-yl)benzene **1f** (1.45 mL, 10.0 mmol) to deliver carboxylic acid **3f** as a colorless oil (729 mg, 4.49 mmol, 58% yield). Spectral data matched that previously reported.[10]

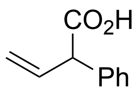

3f

2-Vinyldodecanoic acid (3g): Prepared according to the representative procedure using (*E*)-1-bromotridec-2-ene **1g** (2.50 mL, 10.0 mmol) to deliver the carboxylic acid **3g** as a colorless oil (660 mg, 2.92 mmol, 36% yield). Spectral data matched that previously reported.[12]

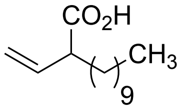

3g

2-methyl-5-(prop-1-en-2-yl)cyclohex-2-ene-1-carboxylic acid (3h): Prepared according to the representative flow procedure above using 6-bromo-1-methyl-4-(prop-1-en-2-yl)cyclohex-1-ene **1h** (2.91 g, 13.5 mmol) to deliver carboxylic acid **3h** as a colorless oil (1.07 g, 5.94 mmol, 50% yield) as an inseparable mixture of diastereomers (1.6:1 dr).

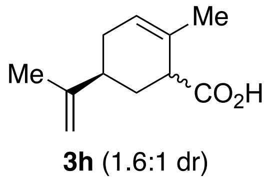

3h (1.6:1 dr)

TLC (30% EtOAc:hexanes, $KMnO_4$), R_f = 0.47

^{1}H NMR (600 MHz, benzene-*d*$_6$, major diastereomer) δ 5.44 (dd, *J* = 5.4, 1.3 Hz, 1H), 4.76 – 4.73 (br s, 1H), 4.73 – 4.69 (br s, 1H), 2.83 (d, *J* = 6.1 Hz, 1H), 2.49 (app td, *J* = 9.8, 5.3 Hz, 1H), 2.14 (ddd, *J* = 13.2, 3.2, 1.8 Hz, 1H), 2.02 (dd, *J* = 17.4, 2.5 Hz, 1H), 1.77 – 1.71 (m, 1H), 1.73 (s, 3H), 1.56 (s, 3H), 1.42 (ddd, *J* = 12.8, 12.7, 6.2 Hz, 1H).

^{13}C NMR (151 MHz, benzene-*d*$_6$, major diastereomer) δ 181.6, 149.0, 129.59, 125.6, 109.3, 45.6, 37.1, 30.9, 30.78, 23.0, 20.9.

^{1}H NMR (600 MHz, benzene-*d*$_6$, minor diastereomer) δ 5.39 (dd, *J* = 4.7, 1.3 Hz, 1H), 4.73 – 4.69 (br s, 1H), 4.69 – 4.65 (br s, 1H), 3.05 (dd, *J* = 8.9, 4.5 Hz, 1H), 1.98 – 1.92 (m, 1H), 1.90 – 1.82 (m, 3H), 1.80 (d, *J* = 11.7 Hz, 1H), 1.69 (s, 3H), 1.51 (s, 3H).

^{13}C NMR (151 MHz, benzene-d_6, minor diastereomer) δ 182.3, 148.7, 129.56, 125.4, 109.7, 48.1, 40.5, 32.3, 30.84, 21.2, 20.5.

IR (Thin Film): 3087, 2971, 2918, 1702, 1642 cm^{-1}.

HRMS: (ESI+) calculated m/z for $C_{11}H_{16}NaO_2$ $[M+Na]^+$: 203.1043, found: 203.1042.

Scheme S1. Attempted allylic and propargylic bromide substrates that failed to deliver isolable amounts of carboxylated product.

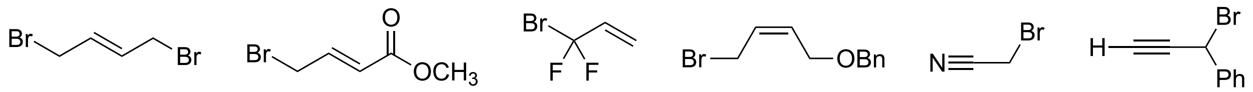

Representative Procedure for *β*-Lactone Synthesis as described for Cyclohexyl-β-lactone 4a

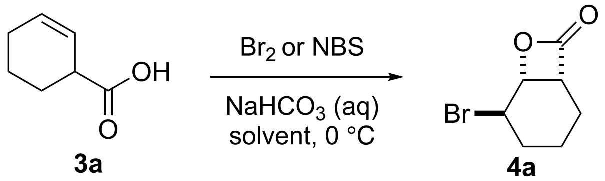

5-bromo-7-oxabicyclo[4.2.0]octan-8-one (4a): Acid **3a** (100 mg taken from a stock solution in Et_2O, 0.79 mmol) was dissolved in a saturated $NaHCO_3$ solution (20 mL) and added slowly to an ice-cold solution of Br_2 (80 μL, 1.5 mmol) in 25 mL Et_2O. The reaction mixture stirred for 1 h at 0 °C, turning off-yellow in color, and was then quenched with a saturated $Na_2S_2O_3$ solution. The organic layer was separated, and the aqueous layer was extracted with diethyl ether (2 x 25 mL). Combined organics were dried over $MgSO_4$, filtered and concentrated. The crude product was purified by automated chromatography (12.0 g silica cartridge, 0 to 50% Et_2O:hexanes gradient elution) to afford *β*-lactone **4a** (68 mg, 0.33 mmol, 42%*).* Spectral data matched that previously reported.[10]

6-bromo-8-oxabicyclo[5.2.0]nonan-9-one (4b): Prepared according to the representative procedure using cyclohept-2-ene-1-carboxylic acid **3b** (50 mg, 0.35 mmol) and NBS (95 mg, 0.54 mmol) in CH_2Cl_2 (10 mL). Upon completion (as judged by TLC, ~1 h), the crude product was purified by automated chromatography (4.0 g silica cartridge, 0 → 50% Et_2O:hexanes gradient elution) to afford *β*-lactone **4b** (53 mg, 0.24 mmol, 68% yield) as a colorless, crystalline solid.

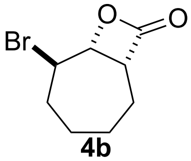

TLC (30% EtOAc:hexanes, $KMnO_4$), R_f = 0.57

VT ^{1}H NMR (400 MHz, benzene-*d*$_6$, 70 °C) δ 4.10 (t, *J* = 7.2 Hz, 1H), 3.78 (t, *J* = 8.6 Hz, 1H), 2.96 – 2.86 (m, 1H), 1.80 (ddd, *J* = 12.3, 6.2, 3.8 Hz, 1H), 1.48 – 1.10 (m, 7H).

^{13}C NMR (151 MHz, $CDCl_3$) δ 176.7, 81.7, 46.3, 43.3, 30.8, 27.9, 24.0, 21.7.

IR (Thin Film): 2932, 2859, 1823, 1454 cm^{-1}.

HRMS: (ESI+) calculated m/z for $C_8H_{12}BrO_2$ $[M+H]^+$: 219.0015, found: 219.0018.

9-bromo-7-oxabicyclo[4.2.1]nonan-8-one (4b'): Prepared according to the representative procedure using cyclohept-2-ene-1-carboxylic acid **3b** (100 mg, 0.71 mmol), and Br_2 (60 μL, 1.1 mmol). Upon completion (as judged by TLC, ~1 h), the crude product was purified by automated chromatography (12.0 g silica cartridge, 0 → 50% Et_2O:hexanes gradient elution) to afford bridged lactone **4b'** (46 mg, 0.43 mmol, ~60% yield, ~90% purity). Note: While minor products could not be isolated in sufficient quantities to determine their structure fully, the IR indicates the presence of a *β*-lactone which could be formed by pathways indicated below that are diastereomeric to β-lactone **4b**.

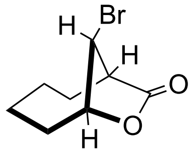

4b'

TLC (10% EtOAc:hexanes, $KMnO_4$), R_f = 0.25

^{1}H NMR (600 MHz, $CDCl_3$) δ 5.04 (td, *J* = 3.4, 0.7 Hz, 1H), 4.39 (s, 1H), 3.13 (dd, *J* = 6.0, 3.3 Hz, 1H), 2.04 (m, 1H), 2.01 – 1.90 (m, 2H), 1.80 (m, 1H), 1.69 – 1.61 (m, 1H), 1.62 – 1.56 (m, 3H).

^{13}C NMR (151 MHz, $CDCl_3$) δ 178.2, 87.6, 51.1, 48.5, 33.0, 30,0, 24.1, 22.2.

IR (Thin Film): 2929, 2864, 1828, 1770 cm^{-1}.

HRMS: (ESI+) calculated m/z for $C_8H_{12}BrO_2$ $[M+H]^+$: 219.0015, found: 219.0017.

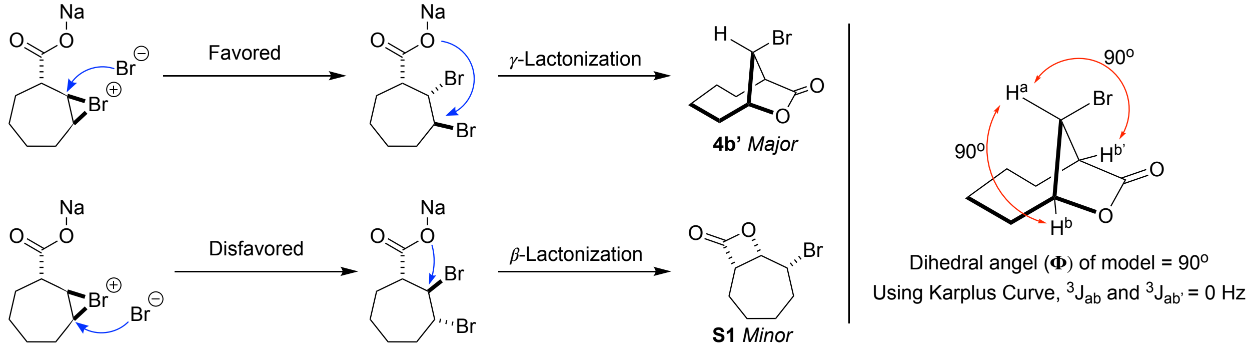

4-(bromomethyl)oxetan-2-one (4e): Prepared according to the representative procedure using but-3-enoic acid **3e** (600 mg, 7.03 mmol) and Br_2 (540 μL, 10.6 mmol). Upon completion (as judged by TLC, ~1 h), the crude product was purified by automated chromatography (12.0 g silica cartridge, 0 → 50% Et_2O:hexanes gradient elution) to afford *β*-lactone **4e** (367 mg, 2.22 mmol, 32% yield). Spectral data matched that previously reported.[10]

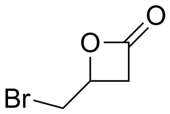

4e

4-(bromomethyl)-3-phenyloxetan-2-one (4f/4f'): Prepared according to the representative procedure using 2-phenylbut-3-enoic acid **3f** (683 mg, 4.21 mmol) and Br_2 (330 μL, 6.32 mmol). Upon completion (as judged by TLC, ~1 h), the crude product was purified by automated chromatography (12.0 g silica cartridge, 0 → 50% Et_2O:hexanes gradient elution) to afford *β*-lactones **4f/4f'** (475 mg, 1.98 mmol, 47% yield) as an inseparable mixture of diastereomers (3:1 dr). Spectral data matched that previously reported.[10]

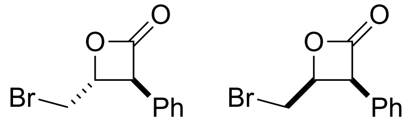

4f **4f'**

4-(bromomethyl)-3-decyloxetan-2-one (4g/4g'): Prepared according to the representative procedure using 2-vinyldodecanoic acid **3g** (118 mg, 0.521 mmol) and NBS (121 mg, 0.680 mmol). Upon completion (as judged by TLC, ~1 h), the crude product was purified by automated chromatography (12.0 g silica cartridge, 0 → 50% Et_2O:hexanes gradient elution) to afford *β*-lactone **4g/4g'** (120 mg, 0.396 mmol, 76% yield) as an inseparable mixture of diastereomers (6:1 dr).

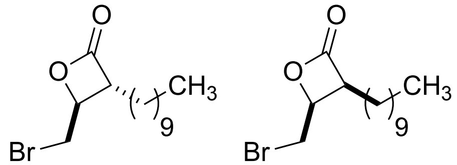

4g **4g'**

TLC (30% Et_2O:hexanes, $KMnO_4$), R_f = 0.75

^{1}H NMR (600 MHz, $CDCl_3$) δ 4.42 (ddd, *J* = 7.6, 4.9, 3.8 Hz, 1H), 3.70 (dd, *J* = 10.8, 5.0 Hz, 1H), 3.51 (dd, *J* = 10.8, 7.6 Hz, 1H), 3.43 (ddd, *J* = 8.5, 6.8, 3.8 Hz, 1H), 1.90 – 1.84 (m, 1H), 1.82 – 1.74 (m, 1H), 1.52 – 1.40 (m, 2H), 1.34 – 1.22 (m, 14H), 0.88 (t, *J* = 7.0 Hz, 3H).

^{13}C NMR (151 MHz, $CDCl_3$) δ 169.8, 74.7, 57.0, 32.0, 31.4, 29.68, 29.63, 29.42, 29.40, 29.3, 27.9, 27.0, 22.8, 14.3.

IR (Thin Film): 2925, 2854, 1832, 1465 cm^{-1}.

HRMS: (ESI+) calculated m/z for $C_{14}H_{26}BrO_2$ $[M+H]^+$: 305.1111, found: 305.1112.

Buta-2,3-dienoic acid (6a), and but-3-ynoic acid (7a):

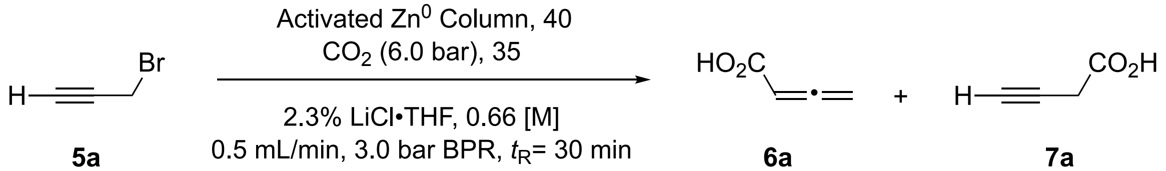

5a **6a** **7a**

Prepared according to the general procedure for conversion of allylic halides to β,γ-unsaturated acid described for acid **3a** (*vide supra*). An oven-dried 25 mL pear-shaped flask was charged with bromide **5a** (750 μL, 10.0 mmol; passed through a plug of alumina until colorless), then dissolved in 15 mL of a 2.3% LiCl•THF solution. This solution was drawn into the flow reactor. Following acid-base extraction and concentration this gave a mixture of the allene acid **6a** and propargylic acid **7a** as a colorless oil (237 mg, 2.82 mmol, 33% yield) and as an inseparable 4:1 mixture of allene and alkyne constitutional isomers. Spectral data matched that previously reported.[13]

buta-2,3-dienoic acid (6b), and but-3-ynoic acid (7b): Prepared according to the representative procedure using (3-bromoprop-1-yn-1-yl)trimethylsilane **5b** (750 μL, 5.30 mmol). Following the acid-base extraction procedure and concentration, this gave a mixture of the carboxylic acids **6b** and **7b** as a colorless oil (132 mg, 1.58 mmol, 38% yield) and as a 13:1 inseparable mixture of allene and alkyne constitutional isomers. Spectral data matched that previously reported.[13]

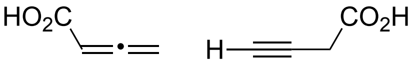

6b **7b**

2-methylbuta-2,3-dienoic acid (6c): Prepared according to the representative procedure using 1-bromobut-2-yne **5c** (900 μL, 10.0 mmol). Following the acid-base extraction procedure and concentration, this gave the carboxylic acid **6c** as a colorless solid (435 mg, 4.43 mmol, 53% yield). Spectral data matched that previously reported.[14]

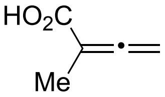

6c

2-phenylbuta-2,3-dienoic acid (6d), and 4-phenylbut-3-ynoic acid (7d): Prepared

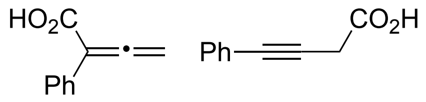

6d **7d**

according to the representative procedure using (3-bromoprop-1-yn-1-yl)benzene **5d** (700 μL, 4.85 mmol). Following the acid-base extraction procedure and concentration delivered the carboxylic acids **6d** and **7d** as a colorless oil (220 mg, 1.37 mmol, 30% yield) in a 2:1 inseparable mixture of allene and alkyne constitutional isomers. Spectral data matched that previously reported.[15]

nona-2,3-dienoic acid (6e), and 2-ethynylheptanoic acid (7e): Prepared according to

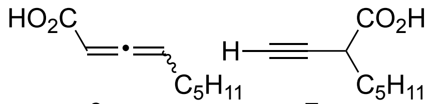

6e **7e**

the representative procedure using 3-bromooct-1-yne **5e** (950 μL, 6.00 mmol). Following the acid-base extraction procedure and concentration delivered the carboxylic acids **6e** and **7e** as a yellow oil (246 mg, 1.60 mmol, 34% yield) in a 1:3 inseparable mixture of allene and alkyne constitutional isomers. Spectral data for allene **6e** matched that previously reported.[16] Alkyne **7e** previously was previously reported but without spectral data.[17] Data for the mixture of alkyne **7e** and allene **6e** are provided below with non-overlapping peaks identified.

TLC (50% EtOAc:hexanes, $KMnO_4$), R_f = 0.36

^{1}H NMR (600 MHz, $CDCl_3$) δ 9.99 (br s, 1H, alkyne/allene), 5.68 (td, *J* = 7.0, 6.1 Hz, 1H, allene), 5.58 (dt, *J* = 6.1, 3.0 Hz, 1H, allene), 3.39 (ddd, *J* = 8.3, 5.9, 2.6 Hz, 1H, alkyne), 2.29 (d, *J* = 2.5 Hz, 1H, alkyne), 2.15 (qd, *J* = 7.2, 3.0 Hz, 2H, allene), 1.90 – 1.77 (m, 2H, alkyne), 1.55 – 1.43 (m, 2H, alkyne/allene), 1.37 – 1.26 (m, 4H, alkyne/allene), 0.89 (t, *J* = 6.9 Hz, 3H, alkyne/allene).

^{13}C NMR (151 MHz, $CDCl_3$, for alkyne **7e**) δ 177.1, 79.9, 72.4, 38.0, 32.3, 31.3, 26.6, 22.52, 14.10.

^{13}C NMR (151 MHz, $CDCl_3$, for allene **6e**) δ 214.0, 172.5, 96.0, 87.9, 31.2, 28.5, 27.4, 22.49, 14.13.

IR (Thin Film): 3310, 2931, 2861, 1958, 1707 cm^{-1}.

HRMS: (ESI+) calculated m/z for $C_9H_{15}O_2$ $[M+H]^+$: 155.1067, found: 155.1067.

2-phenylpenta-2,3-dienoic acid (6f), and 2-methyl-4-phenylbut-3-ynoic acid (7f):

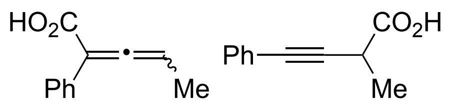

6f **7f**

Prepared according to the representative procedure using (3-bromobut-1-yn-1-yl)benzene **5f** (1.40 mL, 8.95 mmol). Following acid-base extraction and concentration, the allenic acid **6f** and propargylic acid **7f** were obtained as a yellow oil (614 mg, 3.53 mmol, 50% yield) as an inseparable mixture (1:1.5) of allene and alkyne constitutional isomers. Spectral data for propargylic acid **7f** matched that previously reported.[18] However, allene **6f** was previously unreported, thus spectral data is included below and was extracted from NMR. Data for the mixture of alkyne **7e** and allene **6e** are provided below with non-overlapping peaks identified.

TLC (30% EtOAc:hexanes, UV), R_f = 0.31

^{1}H NMR (600 MHz, $CDCl_3$) δ 7.45 – 7.43 (m, 1H, alkyne/allene), 7.36 – 7.25 (m, 4H, alkyne/allene), 6.56 (q, *J* = 2.9 Hz, 1H, allene), 3.69 (q, *J* = 7.2 Hz, 1H, alkyne), 2.00 (d, *J* = 2.9 Hz, 3H, allene), 1.58 (d, *J* = 7.2 Hz, 3H, alkyne).

^{13}C NMR (151 MHz, $CDCl_3$, for alkyne **6e**) δ 177.5, 131.92, 128.40, 128.35, 122.9, 86.3, 83.3, 33.0, 18.2.

^{13}C NMR (151 MHz, $CDCl_3$, for allene **6f**) δ 213.8, 172.4, 131.86, 129.0, 128.1, 127.6, 99.1, 97.9, 14.9.

IR (Thin Film): 2989, 1944, 1702, 1601, 1490 cm^{-1}.

HRMS: (ESI+) calculated m/z for $C_{11}H_{10}NaO_2$ $[M+Na]^+$: 197.0573, found: 197.0573.

Halo-Lactonization of Propargyl Acid 7e

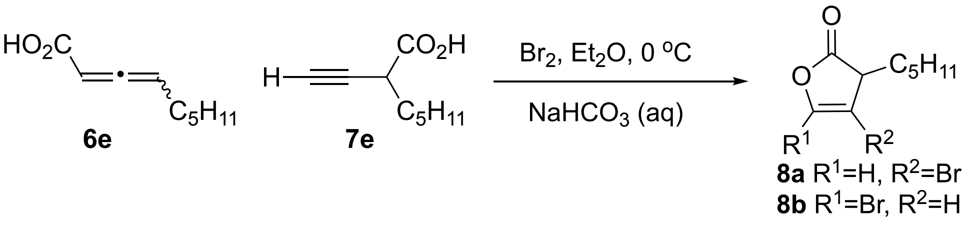

4-bromo-3-pentylfuran-2(3*H*)-one (8a), and 5-bromo-3-pentylfuran-2(3*H*)-one (8b): Carboxylic acid mixture **6e** and **7e** (385 mg, 2.50 mmol) was dissolved in a saturated $NaHCO_3$ solution (15 mL) and added slowly to an ice-cold solution of Br_2 (160 μL, 2.75 mmol) in 15 mL diethyl ether. The reaction mixture stirred for 1 hour at 0 °C, turning off-

yellow in color, and was then quenched with a saturated $Na_2S_2O_3$ solution. The organic layer was separated and the aqueous layer was extracted with diethyl ether (2 x 25 mL). Combined organics were dried over $MgSO_4$, filtered and concentrated. The crude product was purified by automated chromatography (12.0 g silica cartridge, 0 → 50% EtOAc:hexanes gradient elution) to afford furanone **8a** (261 mg, 1.03 mmol, ~41% yield, ~80% purity*)* and furanone **8b** (89 mg, 0.35 mmol, 15%). Note: Minor quantities (~13%) of known butenolide[19] **S2** was isolated but was inseparable from furanone **8a**, see below.

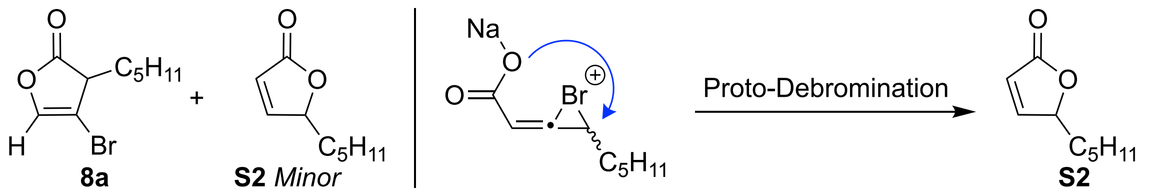

TLC (30% EtOAc:hexanes, $KMnO_4$), R_f = 0.33

¹H NMR (600 MHz, $CDCl_3$) δ 6.70 (s, 1H), 4.11 (dd, *J* = 9.6, 5.3 Hz, 1H), 1.97 – 1.92 (m, 1H), 1.83 – 1.77 (m, 1H), 1.35 – 1.29 (m, 6H), 0.90 (t, *J* = 6.3 Hz, 3H).

¹³C NMR (101 MHz, $CDCl_3$) δ 175.9, 123.3, 106.9, 49.8, 31.5, 29.4, 26.1, 22.5, 14.1.

IR (Thin Film): 2925, 2282, 1711, 1408 cm^{-1}.

HRMS: (ESI+) calculated m/z for $C_9H_{13}BrNaO_2$ $[M+Na]^+$: 254.9991, found: 254.9990.

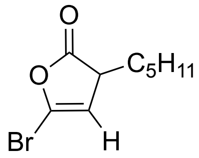

8b

TLC (30% EtOAc:hexanes, $KMnO_4$), R_f = 0.42

¹H NMR (600 MHz, $CDCl_3$) δ 6.30 (d, *J* = 1.7 Hz, 1H), 4.98 (ddd, *J* = 7.6, 3.5, 1.7 Hz, 1H), 2.08 – 2.01 (m, 1H), 1.67 – 1.60 (m, 1H), 1.46 – 1.39 (m, 2H), 1.36 – 1.30 (m, 4H), 0.91 – 0.88 (m, 3H).

¹³C NMR (101 MHz, $CDCl_3$) δ 170.5, 150.9, 122.3, 85.6, 32.0, 31.4, 23.6, 22.5, 14.1.

IR (Thin Film): 2929, 2348, 1758, 1604 cm^{-1}.

HRMS: (ESI+) calculated m/z for $C_9H_{13}BrNaO_2$ $[M+Na]^+$: 254.9991, found: 254.9990.

References

(1) Berton, M.; Huck, L.; Alcázar, J. On-demand synthesis of organozinc halides under continuous flow conditions. *Nat. Protoc.* **2018**, *13*, 324-334.
(2) Bond, C. W.; Cresswell, A. J.; Davies, S. G.; Fletcher, A. M.; Kurosawa, W.; Lee, J. A.; Roberts, P. M.; Russell, A. J.; Smith, A. D.; Thomson, J. E. Ammonium-Directed Oxidation of Cyclic Allylic and Homoallylic Amines. *J. Org. Chem.* **2009**, *74*, 6735-6748.
(3) Jouanneau, M.; Vellalath, S.; Kang, G.; Romo, D. Natural product derivatization with β-lactones, β-lactams and epoxides toward 'infinite' binders. *Tetrahedron* **2019**, *75*, 3348-3354.
(4) Barrero, A. F.; Herrador, M. M.; Quílez del Moral, J. F.; Arteaga, P.; Arteaga, J. F.; Diéguez, H. R.; Sánchez, E. M. Mild TiIII- and Mn/ZrIV-Catalytic Reductive Coupling of Allylic Halides: Efficient Synthesis of Symmetric Terpenes. *J. Org. Chem.* **2007**, *72* (8), 2988-2995.
(5) Liu, G.; Romo, D. Enantioselective Synthesis of Schulzeines B and C via a β-Lactone-Derived Surrogate for Bishomoserine Aldehyde. *Org. Lett.* **2009**, *11*, 1143-1146.
(6) Yadav, D.; Awasthi, S. K. A Pd NP-confined novel covalent organic polymer for catalytic applications. *New J. Chem.* **2020**, *44*, 1320-1325.
(7) Ishizawa, K.; Majima, S.; Wei, X.-F.; Mitsunuma, H.; Shimizu, Y.; Kanai, M. Copper(I)-Catalyzed Stereodivergent Propargylation of N-Acetyl Mannosamine for Protecting Group Minimal Synthesis of C3-Substituted Sialic Acids. *J. Org. Chem.* **2019**, *84*, 10615-10628.
(8) Okitsu, T.; Sato, K.; Potewar, T. M.; Wada, A. Iodocyclization of Hydroxylamine Derivatives Based on the Control of Oxidative Aromatization Leading to 2,5-Dihydroisoxazoles and Isoxazoles. *J. Org. Chem.* **2011**, *76*, 3438-3449.
(9) Piller, F. M.; Appukkuttan, P.; Gavryushin, A.; Helm, M.; Knochel, P. Convenient preparation of polyfunctional aryl magnesium reagents by a direct magnesium insertion in the presence of LiCl. *Angew. Chem. Int. Ed.* **2008**, *47*, 6802-6806.
(10) Vellalath, S.; Romo, D. Telescoped Synthesis of γ-Bromo-β-Lactones from Allylic Bromides Employing Carbon Dioxide. *Isr. J. Chem.* **2017**, *57*, 335-339.
(11) Ishida, N.; Masuda, Y.; Uemoto, S.; Murakami, M. A Light/Ketone/Copper System for Carboxylation of Allylic C−H Bonds of Alkenes with CO2. *Eur. J. Chem.* **2016**, *22*, 6524-6527.
(12) JoséAurell, M.; Ceita, L.; Mestres, R.; Tortajada, A. Dienediolates of unsaturated carboxylic acids in synthesis. Aldehydes and ketones from alkyl halides, by ozonolysis of β,γ-unsaturated α-alkyl carboxylic acids. The role of a tertiary amine in the cleavage of ozonides. *Tetrahedron* **1997**, *53*, 10883-10898.
(13) Matsushita, K.; Suzuki, K.; Ohmori, K. Total Syntheses of Atrovenetin and Atrovenetinone: A Naphthalene-Annulation Approach to a Discoid Tricycle Using Allenic Acid. *Synlett* **2017**, *28*, 944-950.
(14) Chen, W.; Walker, J. C. L.; Oestreich, M. Metal-Free Transfer Hydroiodination of C–C Multiple Bonds. *J. Am. Chem. Soc.* **2019**, *141*, 1135-1140.
(15) Wei, Y.; Zhao, W.-T.; Yang, Y.-L.; Zhang, Z.; Shi, M. Allenic Esters from Cyclopropenones by Lewis Base Catalysis: Substrate Scope, the Asymmetric Variant from the Dynamic Kinetic Asymmetric Transformation, and Mechanistic Studies. *ChemCatChem* **2015**, *7*, 3340-3349. Fang, W.; Breit, B. Tandem Regioselective Hydroformylation-Hydrogenation of Internal Alkynes Using a Supramolecular Catalyst. *Angew. Chem. Int. Ed.* **2018**, *57*, 14817-14821.

(16) Clinet, J.-C.; Linstrumelle, G. Allenyllithium Reagents; V1. An Efficient Route to Functionalised Allenes. *Synthesis* **1981**, *1981*, 875-878.
(17) Khan, G. R.; Pover, K. A.; Scheinmann, F. Reaction of 1,3-dilithioacetylides with carbonyl electrophiles: preparation of allene-1,3-dicarboxylic acids. *J. Chem. Soc., Chem. Commun.* **1979**, 215-216.
(18) Shen, Y.; Huang, B.; Zheng, J.; Lin, C.; Liu, Y.; Cui, S. Csp–Csp3 Bond Formation via Iron(III)-Promoted Hydroalkynylation of Unactivated Alkenes. *Org. Lett.* **2017**, *19*, 1744-1747.
(19) Nienałtowski, T.; Szczepanik, P.; Małecki, P.; Czajkowska-Szczykowska, D.; Czarnocki, S.; Pawłowska, J.; Kajetanowicz, A.; Grela, K. Large-Scale Synthesis of a Niche Olefin Metathesis Catalyst Bearing an Unsymmetrical N-Heterocyclic Carbene (NHC) Ligand and its Application in a Green Pharmaceutical Context. *Eur. J. Chem.* **2020**, *26*, 15708-15717.
(20) Elamparuthi, E.; Fellay, C.; Neuburger, M.; Gademann, K. Total Synthesis of Cyrneine A. *Angew. Chem. Int. Ed.* **2012**, *51*, 4071-4073.
(21) Zhu, X.; Ganesan, A. Regioselective Synthesis of 3-Alkylindoles Mediated by Zinc Triflate. *J. Org. Chem.* **2002**, *67*, 2705-2708.

^{1}H and ^{13}C NMR Spectra

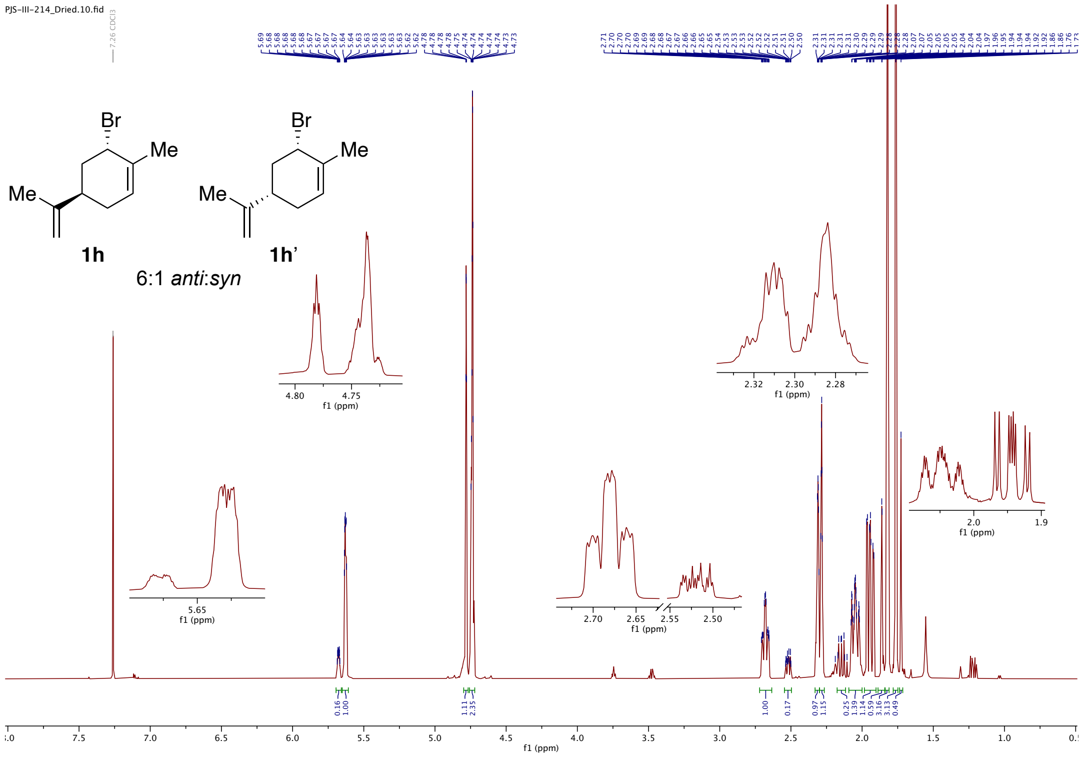

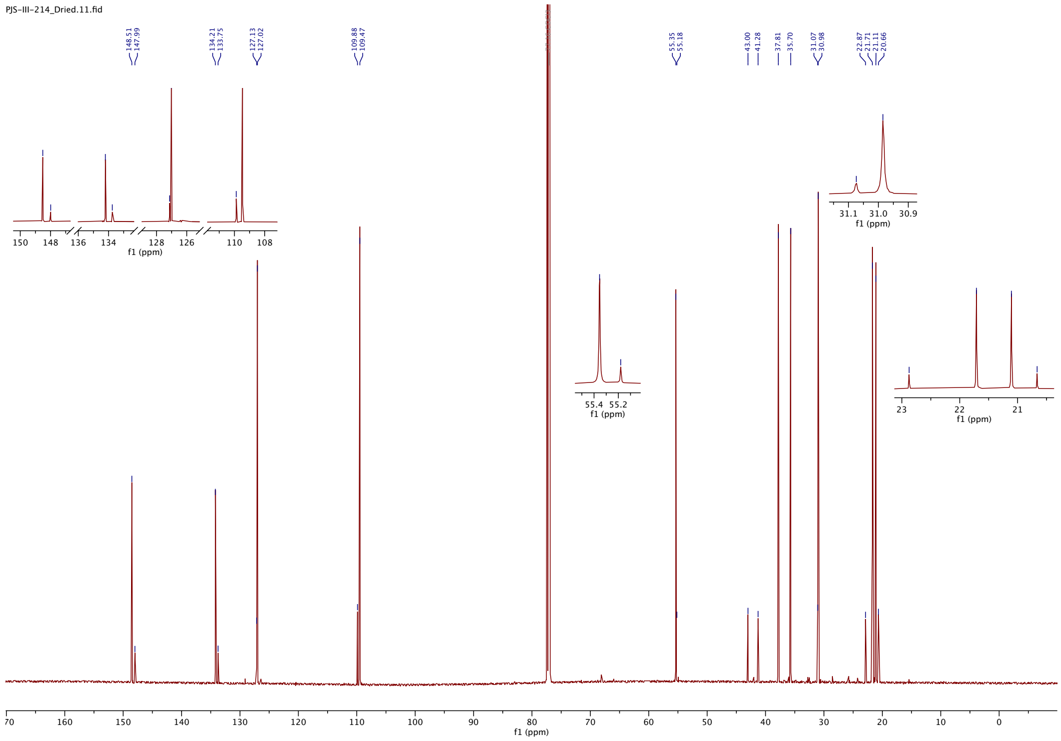

^{1}H (600 MHz) and ^{13}C NMR (151 MHz) spectra for crude material, bromides **1h** and **1h'**, in $CDCl_3$

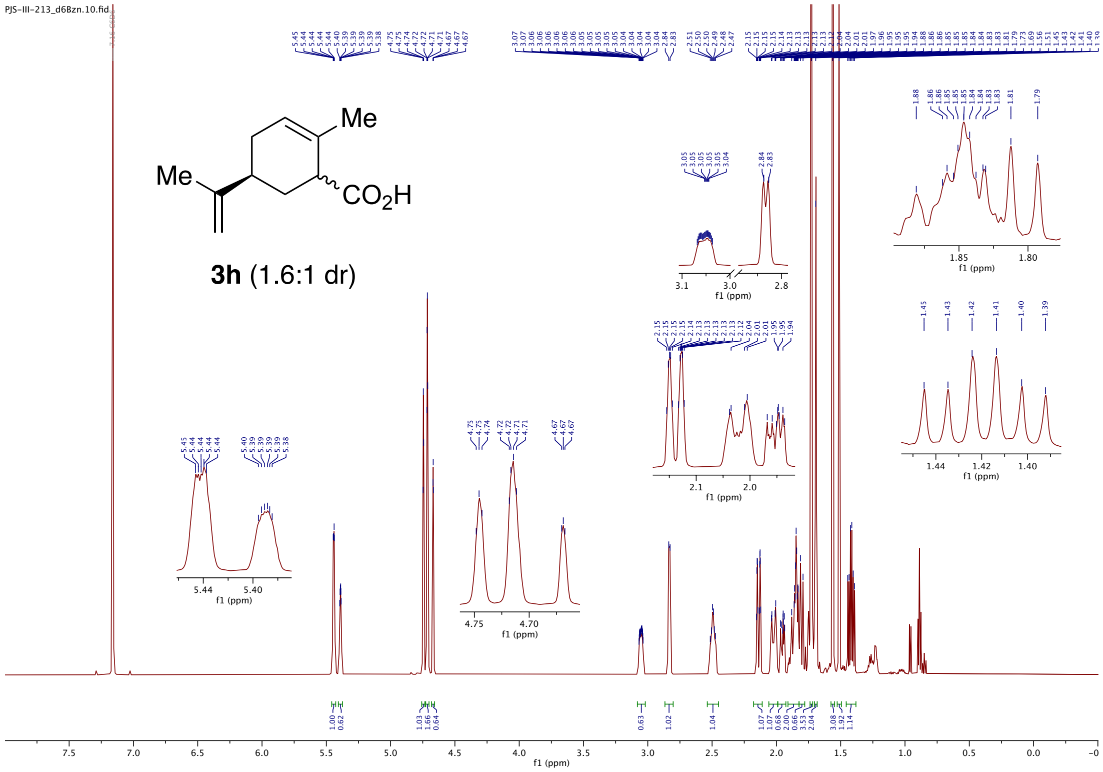

3h (1.6:1 dr)

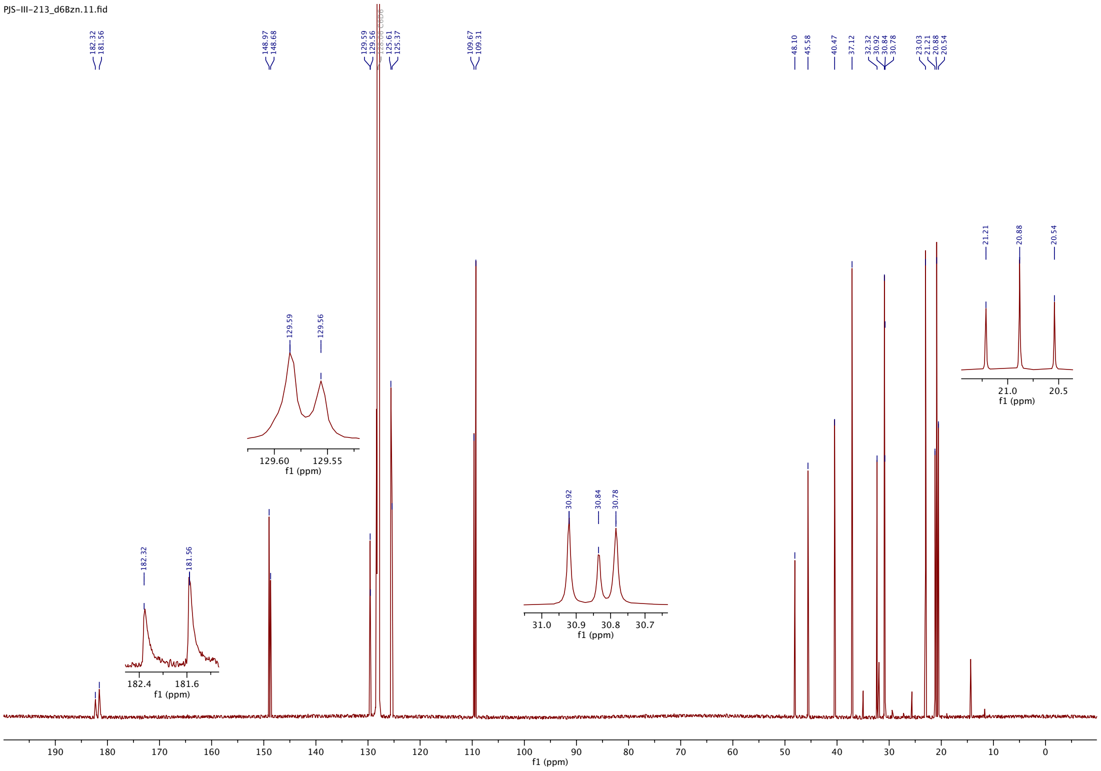

^{1}H (600 MHz) and ^{13}C NMR (151 MHz) spectra for acids **3h** in benzene-d_6

Variable temperature study of β-lactone 4b. This was necessary due to the broad peaks observed at ambient temperature likely due to the multiple slowly interconverting conformers of the 7-membered ring.

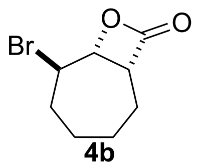

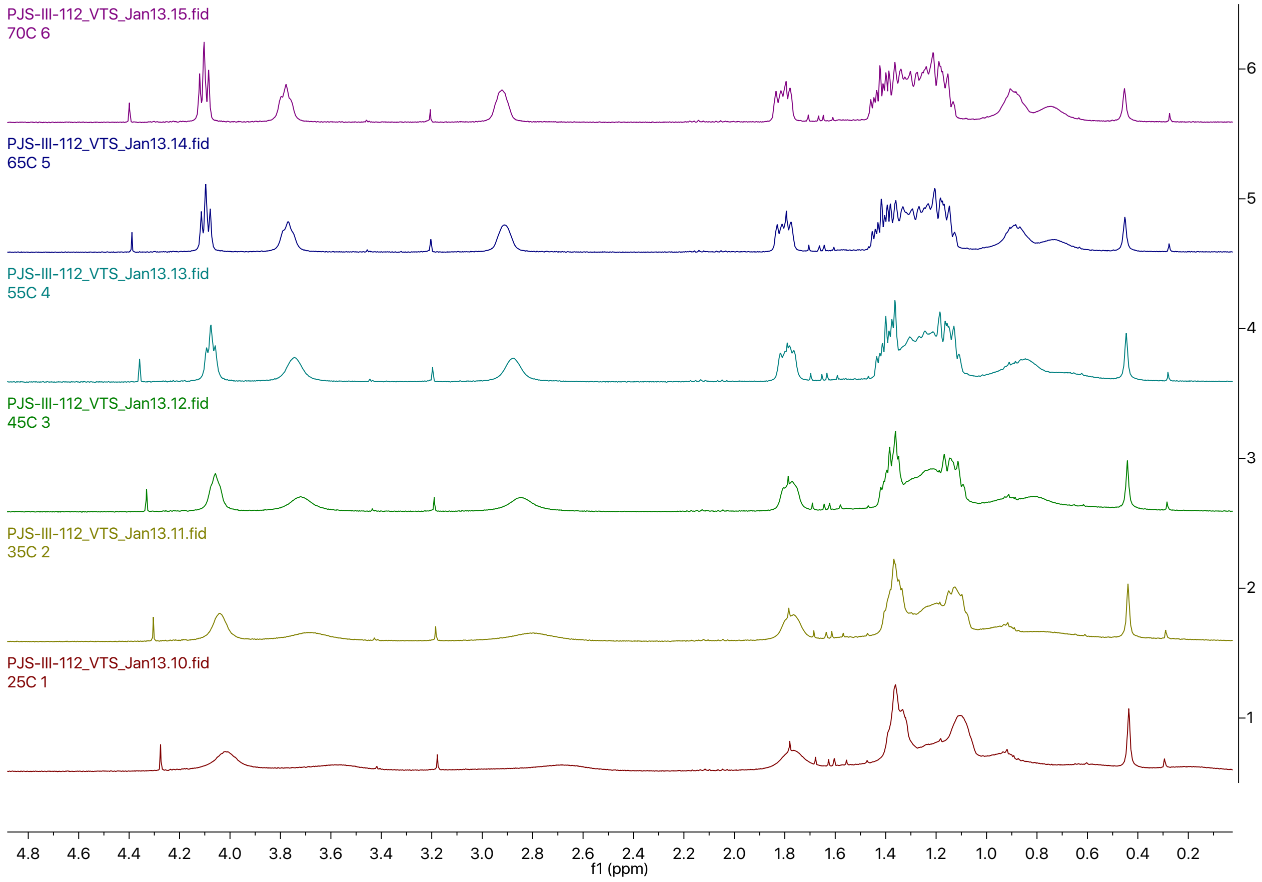

VT ^{1}H NMR of β-lactone **4b** (400 MHz, benzene-d_6, 25 → 70 °C)

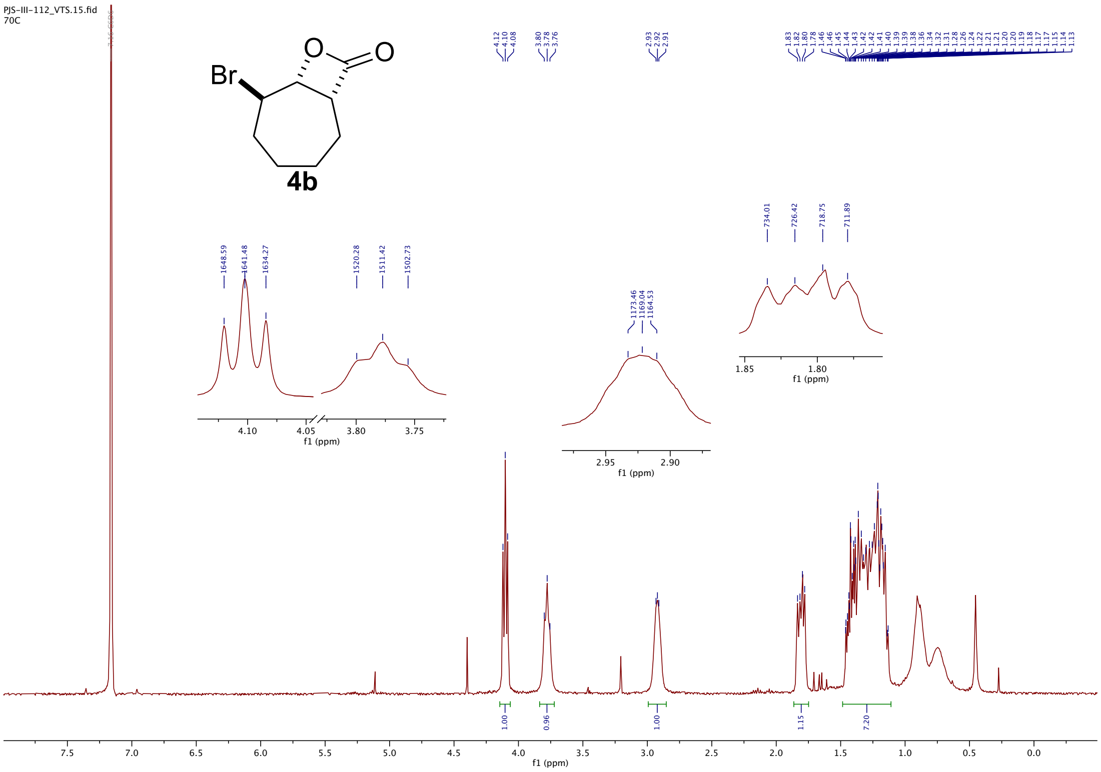

^{1}H (400 MHz, benzene-d_6, 70 °C)

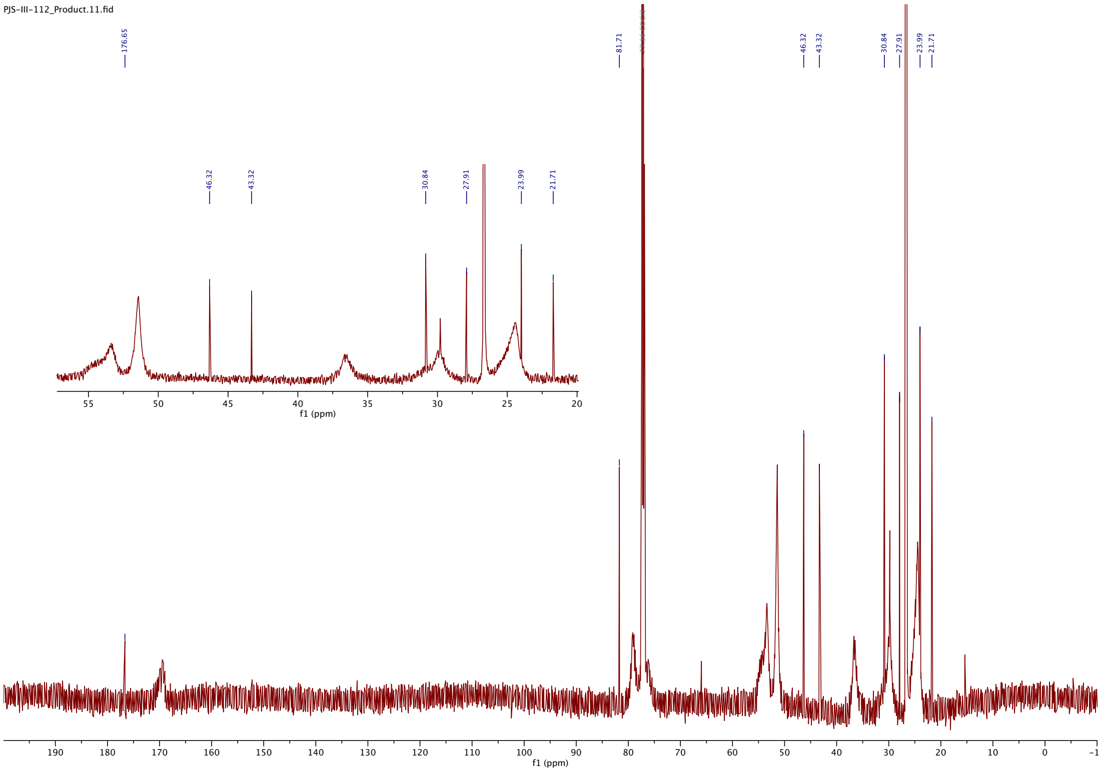

^{13}C NMR (151 MHz, $CDCl_3$) spectra for β-lactone **4b** in benzene-d_6

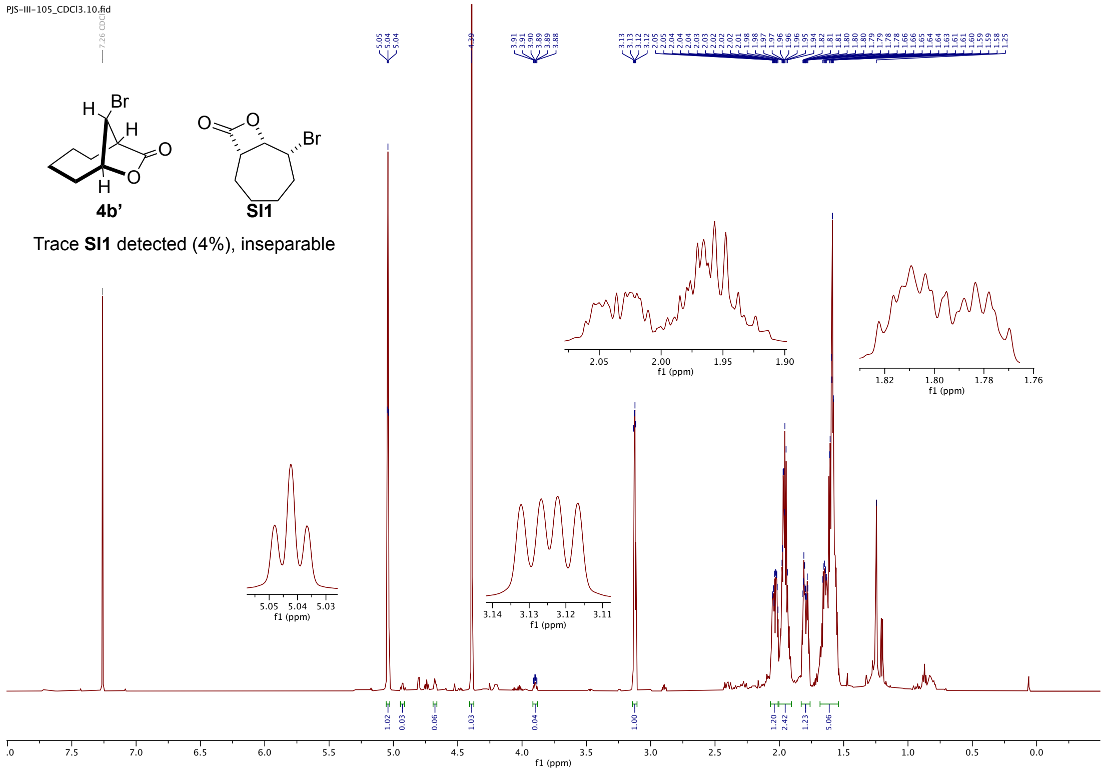

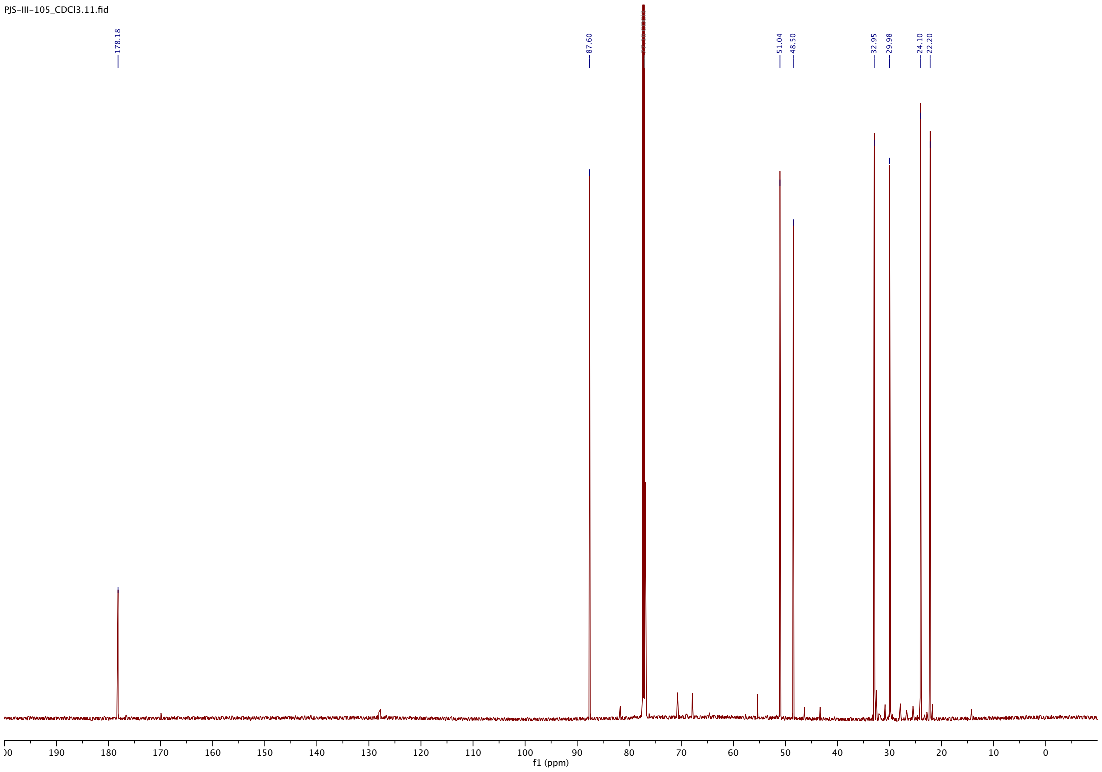

^{1}H (600 MHz) and ^{13}C NMR (151 MHz) spectra for γ-lactone **4b'** in $CDCl_3$

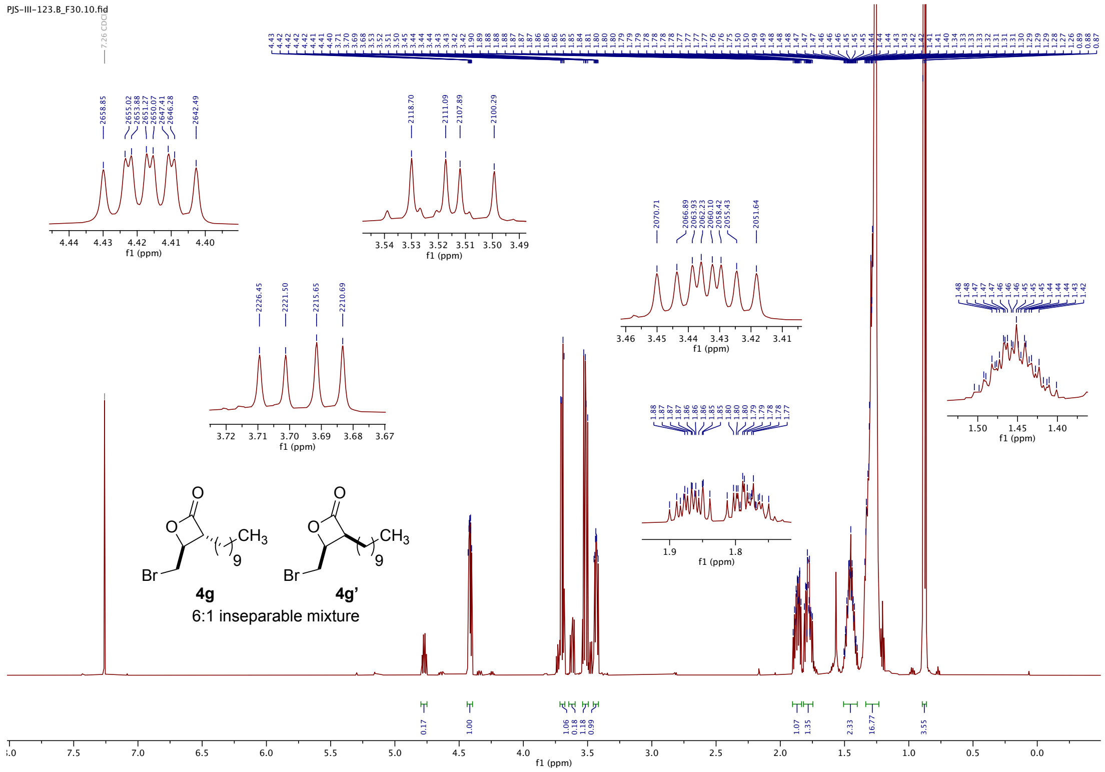

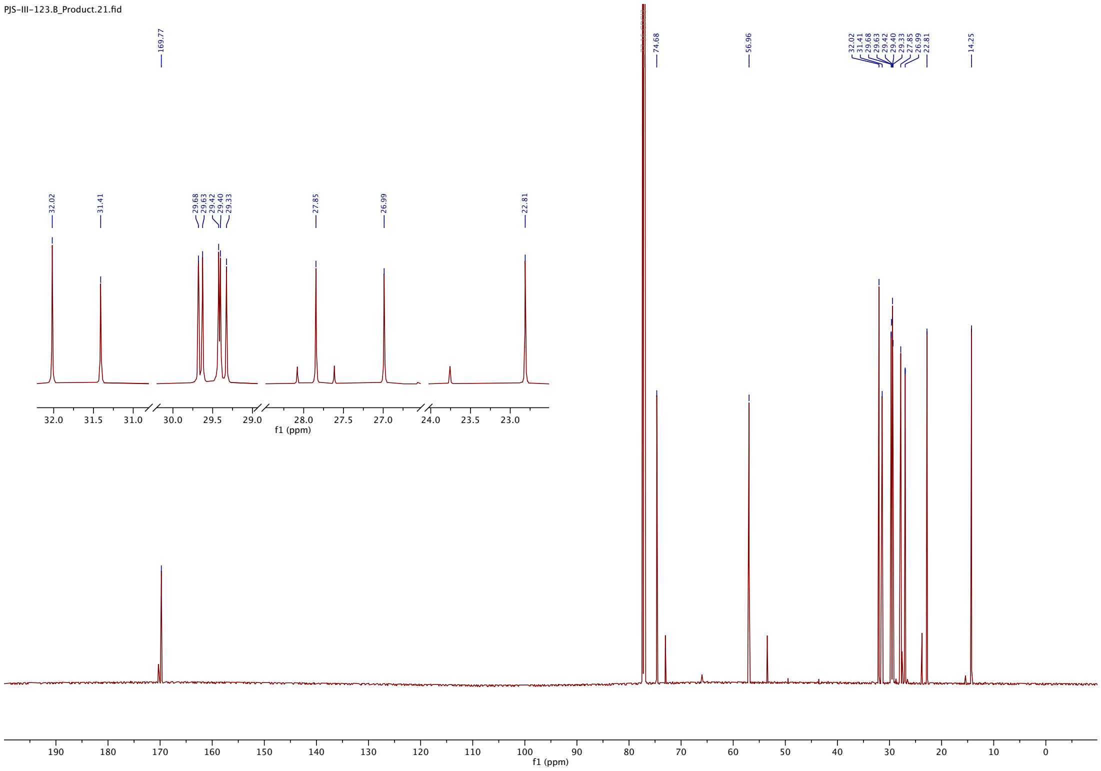

^{1}H (600 MHz) and ^{13}C NMR (151 MHz) spectra for *β*-lactone **4g** in $CDCl_3$

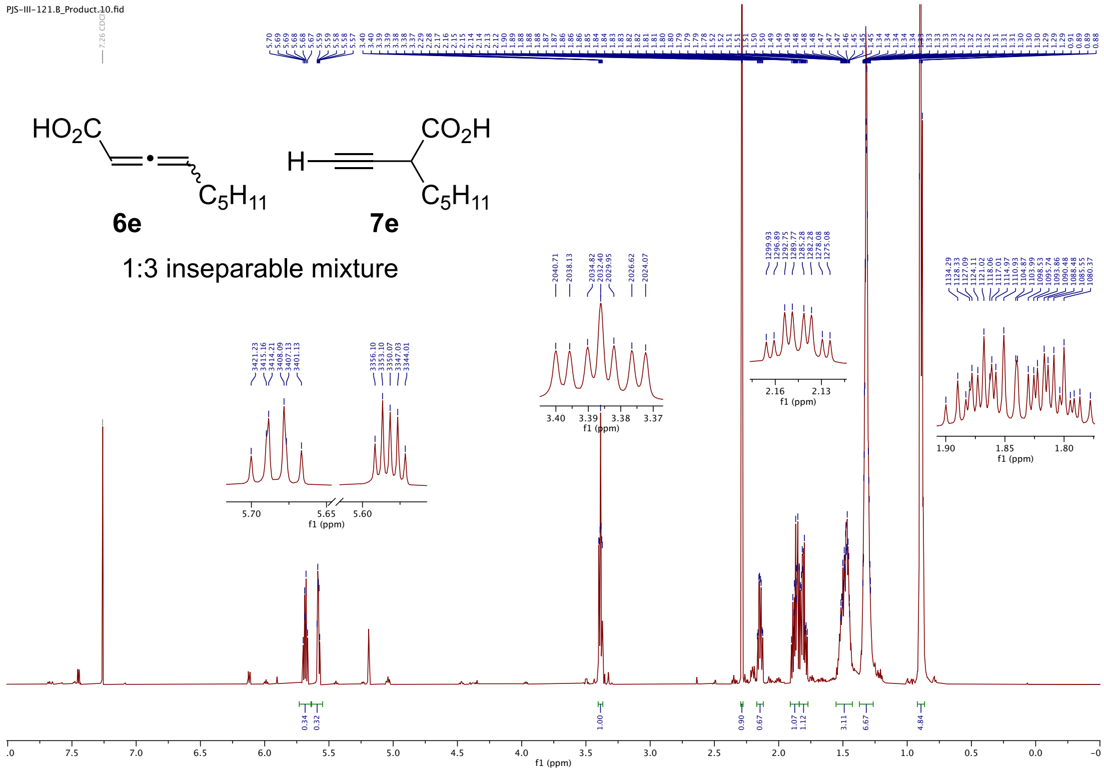

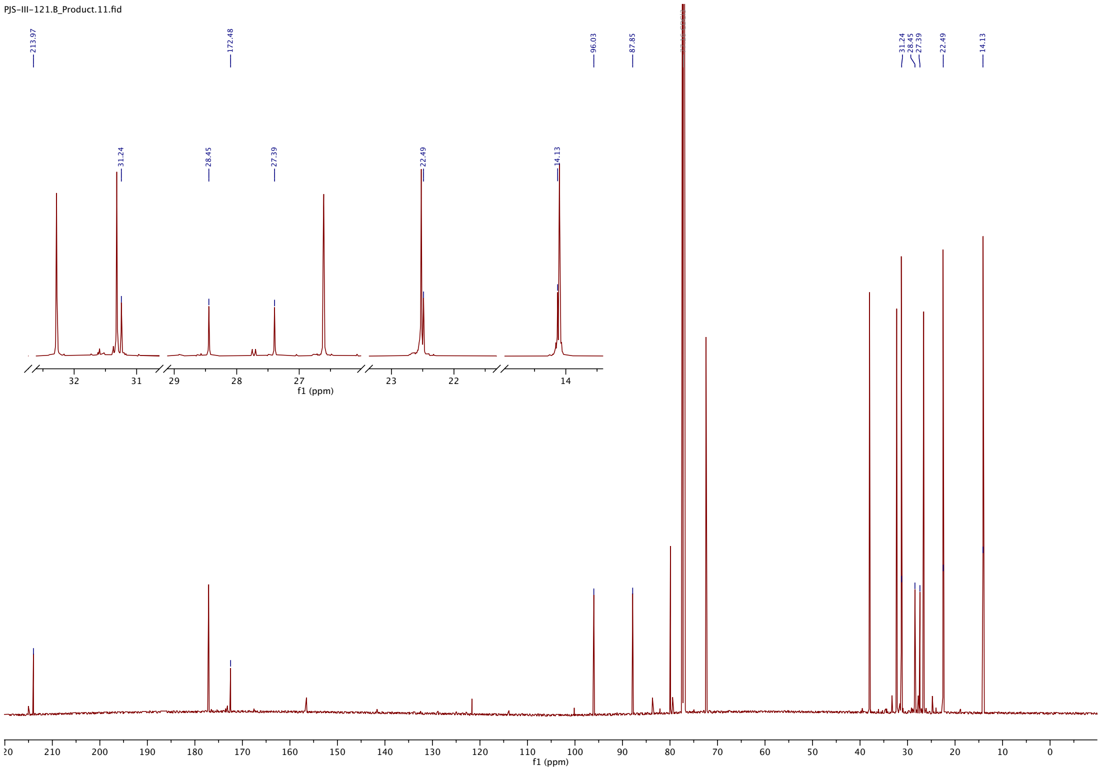

^{1}H (600 MHz) and ^{13}C NMR (151 MHz) spectra for acids **6e** and **7e** in $CDCl_3$

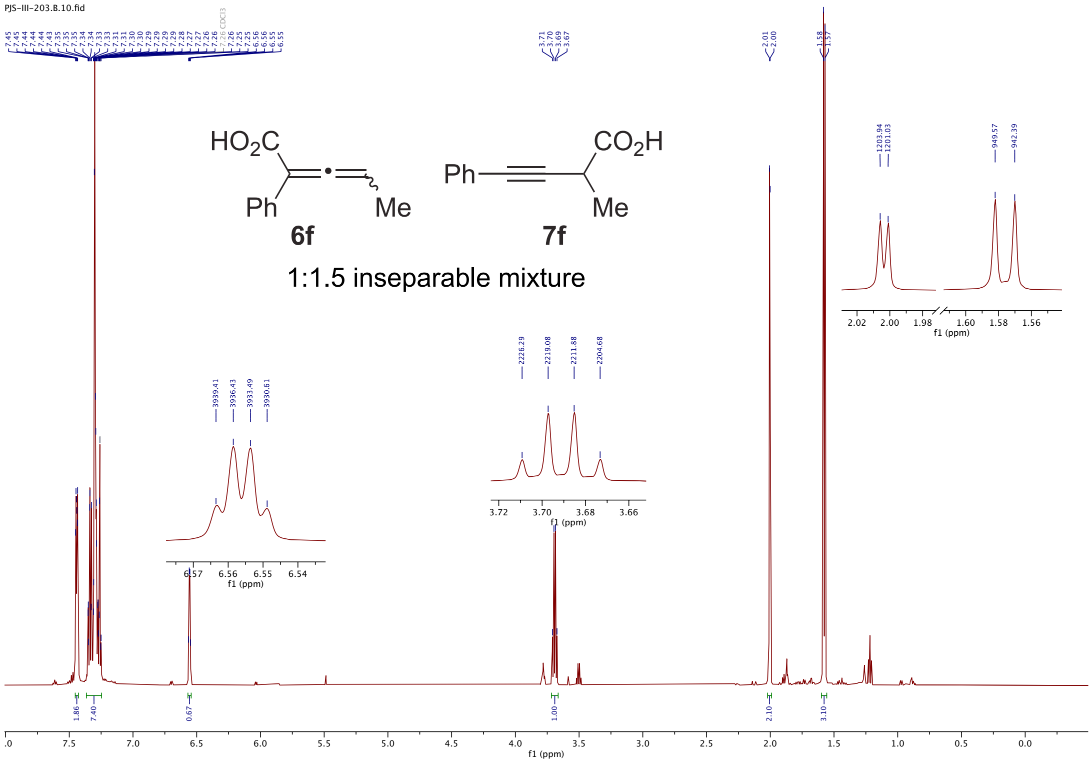

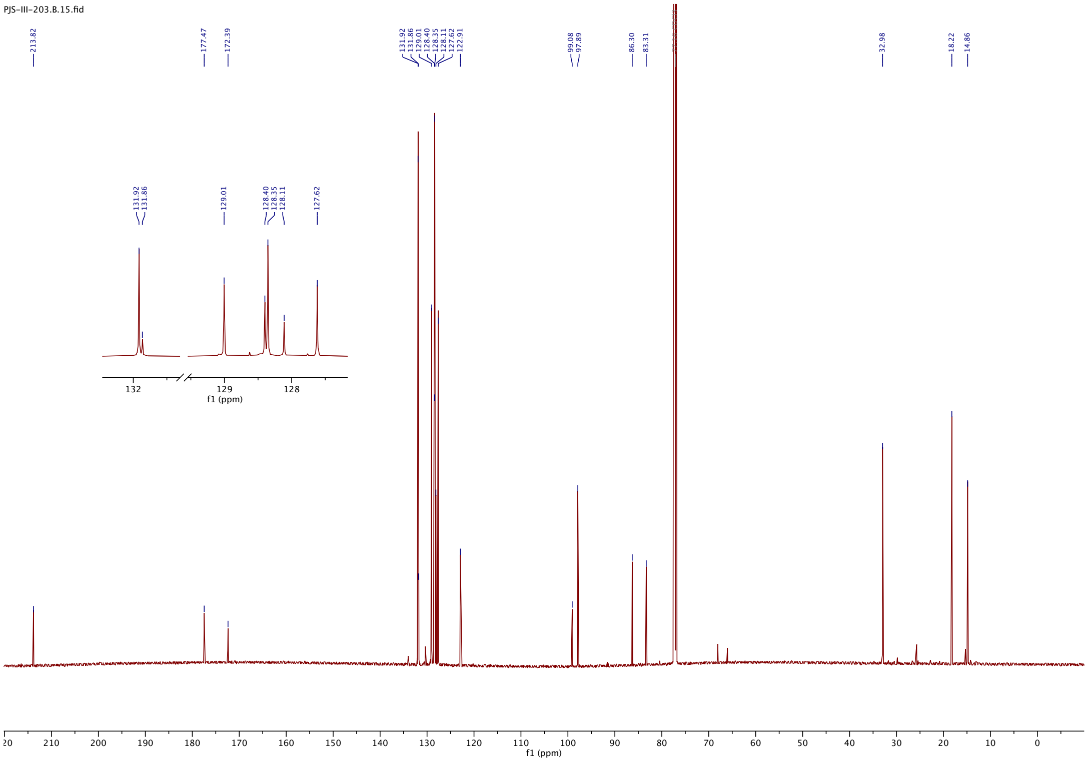

^{1}H (600 MHz) and ^{13}C NMR (151 MHz) spectra for acids **6f** and **7f** in $CDCl_3$

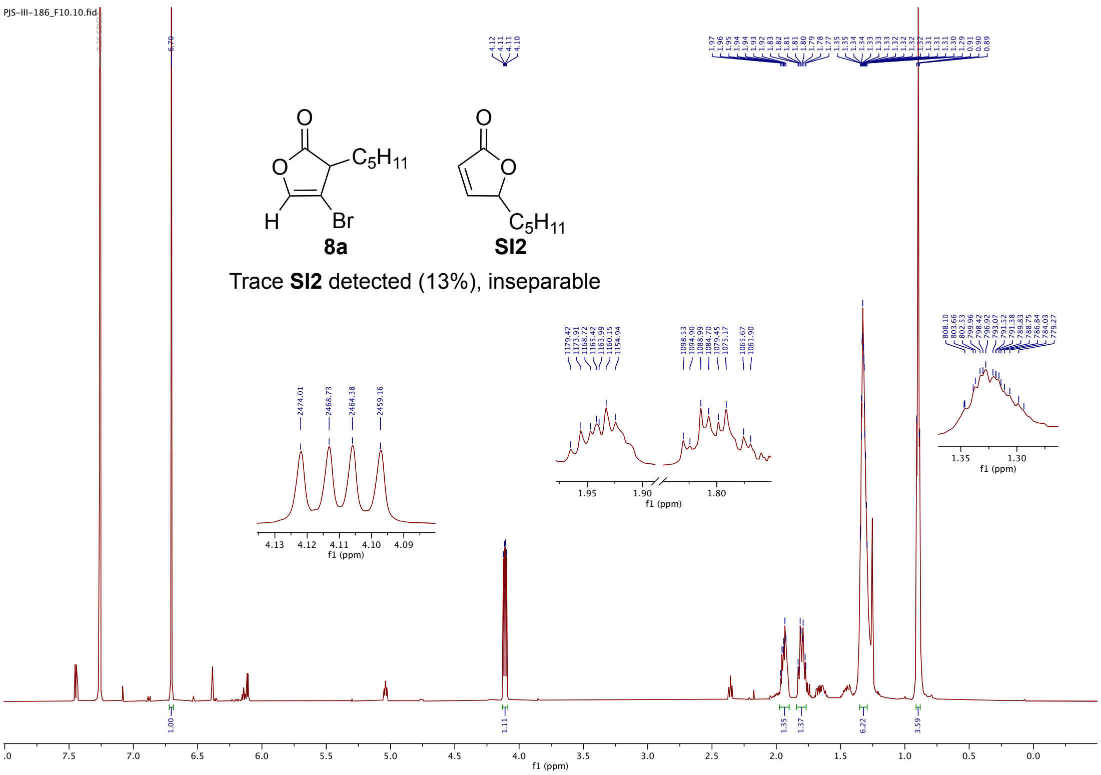

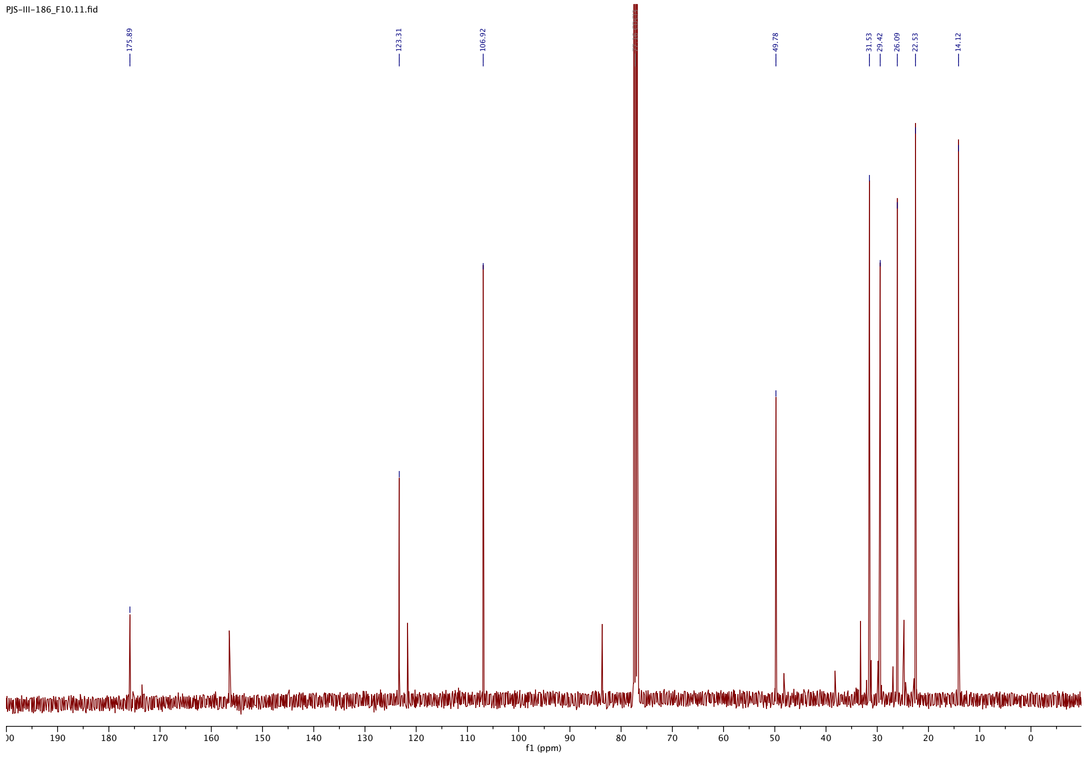

^{1}H (600 MHz) and ^{13}C NMR (151 MHz) spectra for furanone **8a** in $CDCl_3$

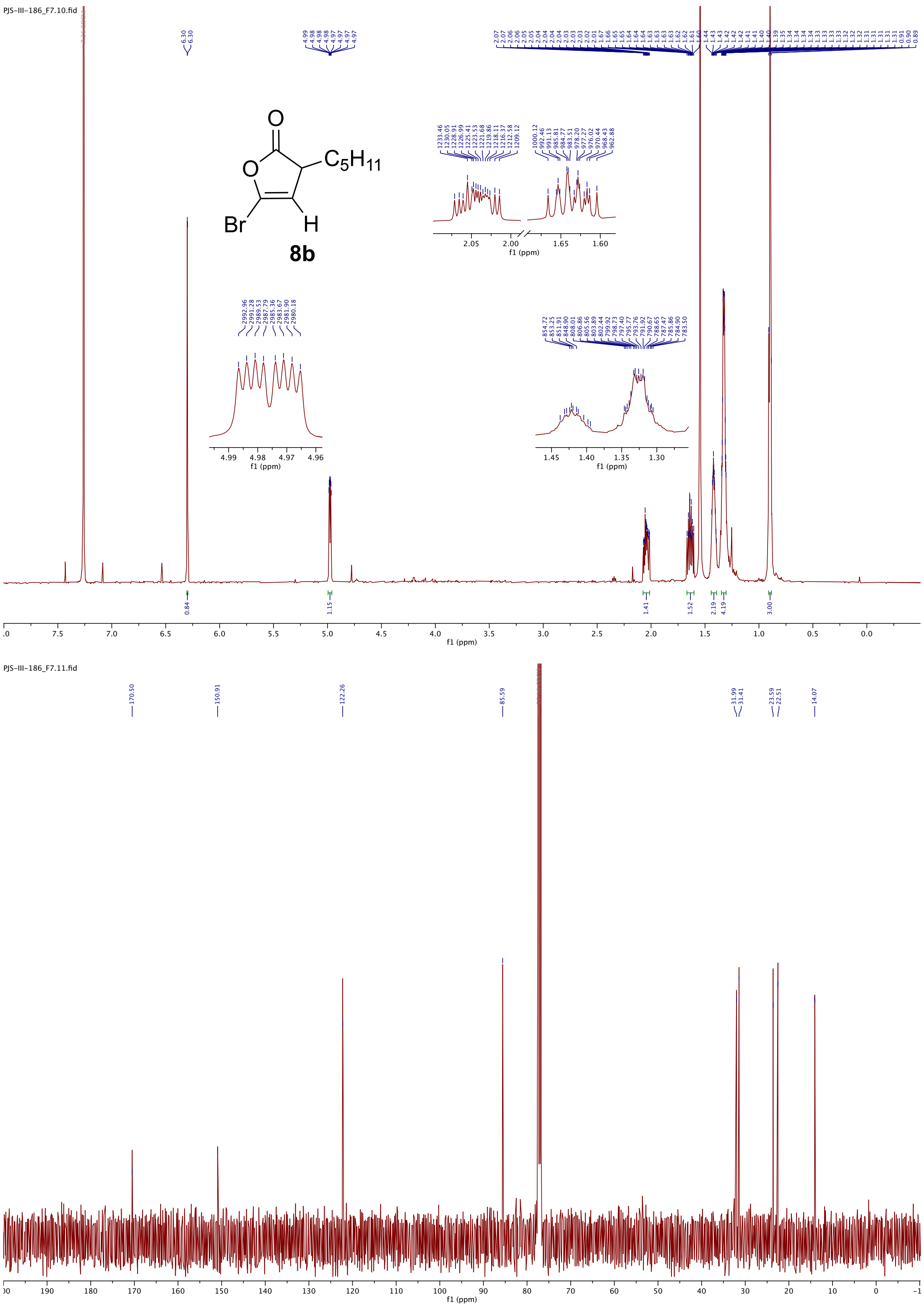

^{1}H (600 MHz) and ^{13}C NMR (151 MHz) spectra for furanone **8b** in $CDCl_3$

X-ray structure of β-lactone 4b and crystallographic data

(CCDC 2142979)

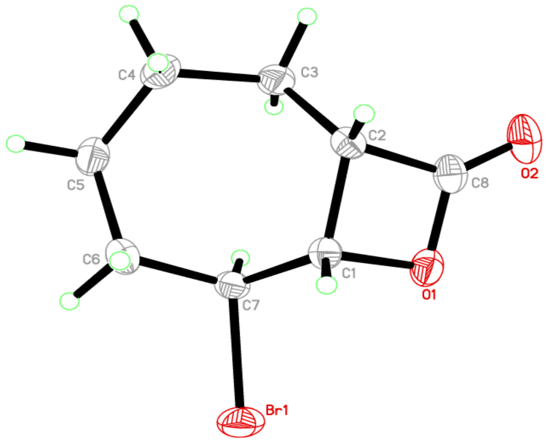

Table S4. X-ray crystallographic data of *β*-lactone **4b**

Empirical formula	$C_8 H_{11} Br O_2$	
Formula weight	219.08	
Temperature	150(2) K	
Wavelength	0.71073 Å	
Crystal system	Monoclinic	
Space group	C2/c	
Unit cell dimensions	a = 25.9184(7) Å	α = 90°.
	b = 5.4615(2) Å	β = 113.6391(10)°.
	c = 13.2419(4) Å	γ = 90°.
Volume	1717.15(9) Å^3	
Z	8	
Density (calculated)	1.695 Mg/m^3	
Absorption coefficient	4.735 mm^{-1}	
F(000)	880	
Crystal size	0.284 x 0.267 x 0.181 mm^3	
Theta range for data collection	3.099 to 28.278°.	
Index ranges	-32<=h<=34, -7<=k<=7, -17<=l<=16	
Reflections collected	15287	
Independent reflections	2123 [R(int) = 0.0370]	
Completeness to theta = 25.242°	99.6 %	

Absorption correction	Semi-empirical from equivalents
Max. and min. transmission	0.293 and 0.212
Refinement method	Full-matrix least-squares on F^2
Data / restraints / parameters	2123 / 0 / 100
Goodness-of-fit on F^2	1.060
Final R indices [I>2sigma(I)]	R1 = 0.0217, wR2 = 0.0533
R indices (all data)	R1 = 0.0224, wR2 = 0.0537
Extinction coefficient	n/a
Largest diff. peak and hole	0.476 and -0.591 e.Å^{-3}

Table S5. Atomic coordinates (x 10^4) and equivalent isotropic displacement parameters ($Å^2$x 10^3) for β-lactone **4b**. U(eq) is defined as one third of the trace of the orthogonalized U^{ij} tensor.

	x	y	z	U(eq)
Br(1)	459(1)	1074(1)	1564(1)	34(1)
C(1)	1503(1)	1285(2)	1385(1)	19(1)
C(2)	1932(1)	1110(2)	847(1)	20(1)
C(3)	1722(1)	1093(2)	-406(1)	24(1)
C(4)	1460(1)	3546(3)	-916(1)	29(1)
C(5)	853(1)	3889(3)	-1036(1)	31(1)
C(6)	745(1)	3874(3)	18(1)	28(1)
C(7)	891(1)	1451(2)	634(1)	21(1)
C(8)	2063(1)	-1364(2)	1412(1)	24(1)
O(1)	1669(1)	-1206(2)	1848(1)	26(1)
O(2)	2371(1)	-3039(2)	1506(1)	35(1)
